# Critical updates on oral insulin drug delivery systems for type 2 diabetes mellitus

**DOI:** 10.1186/s12951-024-03062-7

**Published:** 2025-01-15

**Authors:** Chan Yew Low, Wei Ling Gan, Su Jeat Lai, Rachel Su-May Tam, Jie Fei Tan, Stefanie Dietl, Lay Hong Chuah, Nicolas Voelcker, Athirah Bakhtiar

**Affiliations:** 1 School of Pharmacy, Monash University Malaysia, Jalan Lagoon Selatan, 47500 Bandar Sunway, Selangor Malaysia; 2 Monash Institute of Pharmaceutical Sciences (MIPS), Monash University Parkville Campus, 381 Royal Parade, Parkville, Australia; 3 Department of Pharmacy, University of Copenhagen, Universitetsparken 2, København, Denmark

**Keywords:** Oral insulin, Type 2 diabetes mellitus, Advance drug delivery, Preclinical, Clinical

## Abstract

Type 2 diabetes mellitus (T2DM) is a metabolic disorder characterized by insulin resistance, leading to elevated blood sugar levels. Exogenous insulin can counteract the diminished response to insulin and effectively controlling blood glucose levels, thereby minimizing diabetes-related complications. However, given the injectable nature of exogenous insulin, apprehensions regarding its safety and the difficulties associated with its administration have hindered its widespread and prompt utilization. In this context, advanced oral insulin formulations can improve medication adherence in patients with diabetes and enhance their quality of life. Over the last 20 years, sophisticated pharmaceutical technologies have been utilized to provide insulin through oral formulations. Despite the limited absorption of oral insulin, these studies have demonstrated encouraging outcomes in translating clinical discoveries into commercialization. This review examines the advancements of several oral insulin formulations in preclinical and clinical trials, their effectiveness and safety characteristics, and potential implications for future treatment options.

**Graphical Abstract:**

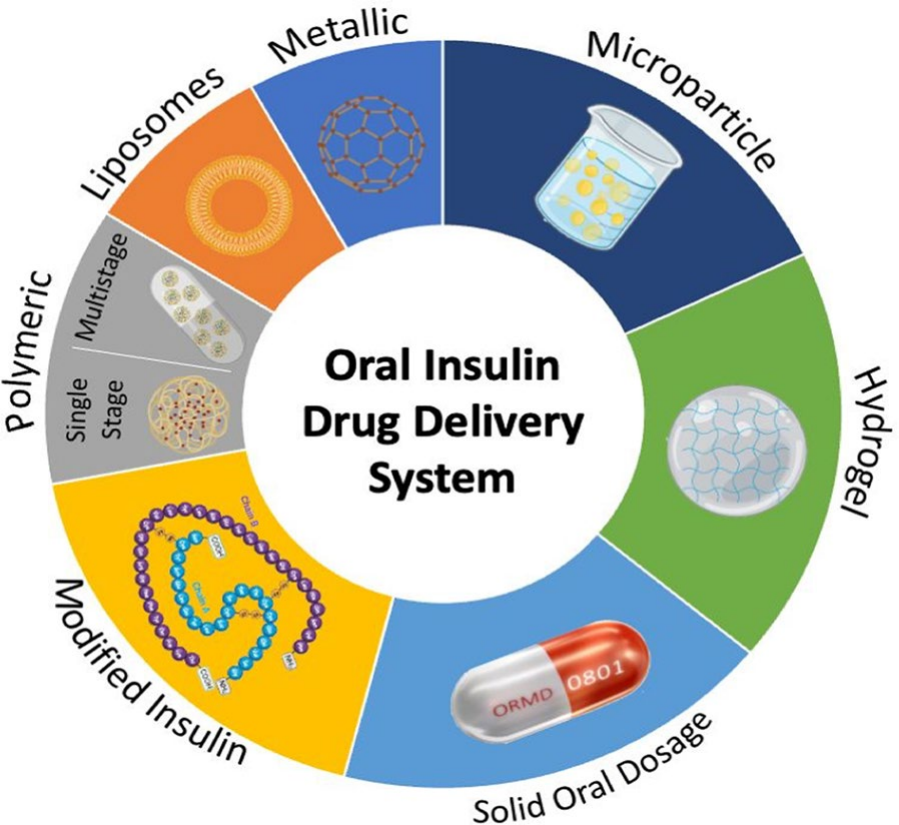

## Introduction

Diabetes mellitus is a chronic metabolic disorder characterized by persistent hyperglycemia caused by insufficient insulin production, the inability of the body to effectively utilize insulin, or a combination of both. These variables can lead to many health repercussions, including cardiovascular diseases, neuropathy, renal failure, and vision loss [1]. Type 2 diabetes mellitus (T2DM) accounts for > 90% of all instances of diabetes mellitus [2]. The primary factors contributing to the development of T2DM are impaired secretion of insulin by pancreatic beta cells and diminished sensitivity of peripheral tissues to insulin, leading to a gradual decline in the production of natural insulin [3]. Exogenous insulin delivery is a potent treatment for severe hyperglycemia or when satisfactory regulation of blood sugar levels cannot be achieved despite the use of different oral hypoglycemic drugs.

**Fig. 1:**
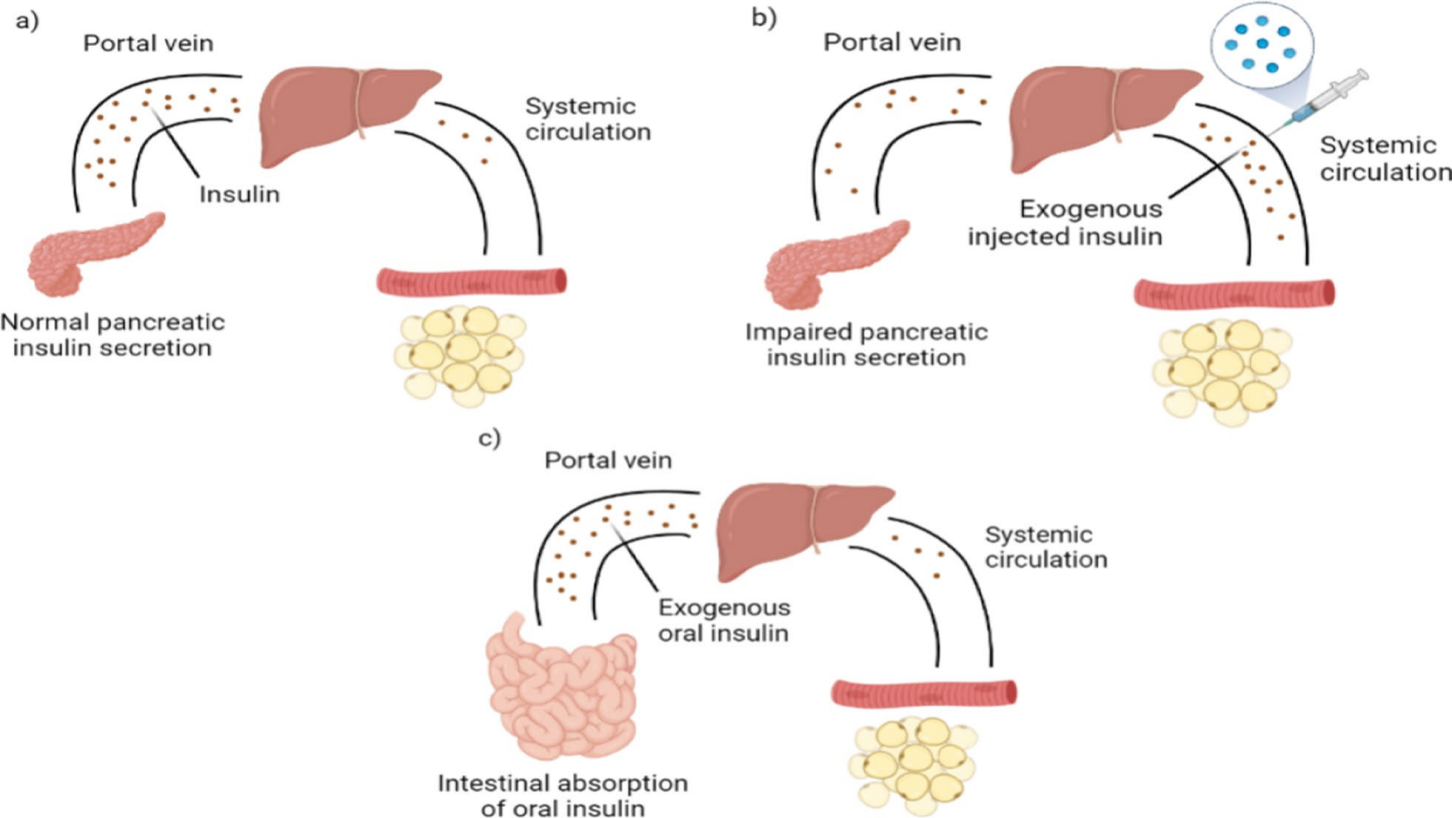
Portal/systemic insulin gradient in normal physiology and after insulin administration. **a** Normal insulin secretion by the pancreatic beta cells results in the uptake of most endogenous insulin by the liver. **b** A low portal/systemic insulin gradient through injection results in lowered glucose suppression in the liver. **c** Oral insulin absorbed in the intestines passes the portal vein and enters the liver, establishing a portal/systemic insulin gradient similar to that observed under normal physiological conditions

However, the traditional subcutaneous (SC) mode of insulin delivery has several limitations, including the intricacy of insulin regimens, potential for hypoglycemia, adverse consequences of weight gain, and requirement for needle puncture. The oral delivery route has, thus, been suggested as an alternative method of administration. This method allows insulin to reach the liver by passing through portal circulation, which creates a gradient of insulin between the liver and the rest of the body, mimicking the natural insulin pathway (Fig. 1) [4]. This enables the inhibition of hepatic gluconeogenesis without any hindrance, while avoiding excessive insulin levels in the peripheral tissues. Elevated peripheral insulin levels are linked to weight gain and low blood sugar levels [5]. In addition, oral administration of insulin is anticipated to enhance patient compliance because of its ease of use, convenience, and non-invasive nature [6].

**Fig. 2:**
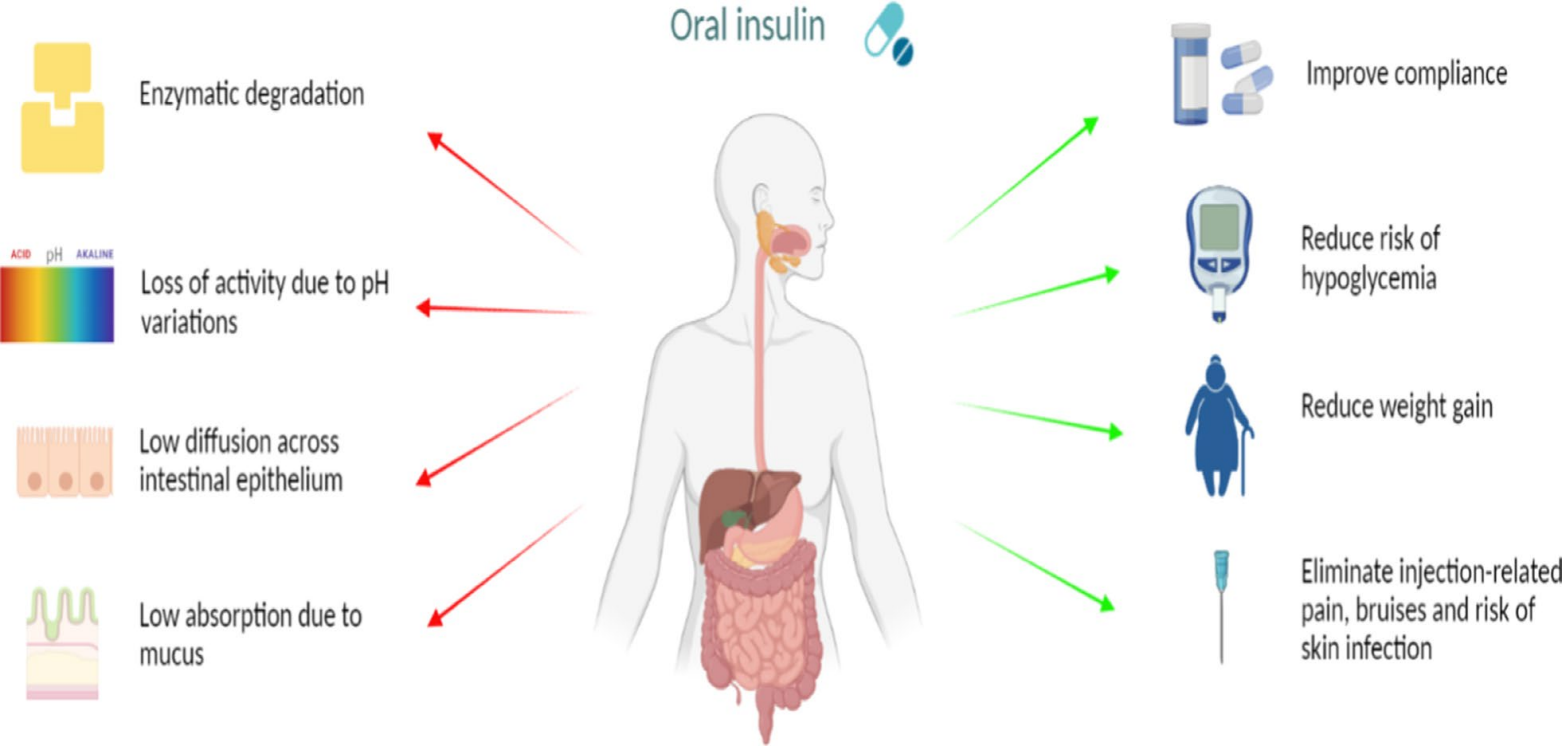
Challenges in developing oral insulin

Attaining effective oral administration of insulin faces notable challenges because of its limited ability to be absorbed by the body, due to physical and chemical hindrances (Fig. 2). The gastrointestinal (GI) tract is lined with an epithelial layer of tightly interconnected cells. This layer acts as a physical barrier preventing the absorption of most peptides [7]. Owing to its high molecular weight (~ 5700 kDa) and hydrophilic properties, insulin has a slow diffusion rate across the epithelial layer [8]. The presence of a mucus barrier within the intestinal epithelium can also hinder the uptake of most medications. In addition, insulin undergoes chemical destruction in the stomach (due to its acidic pH) and enzymatic breakdown in the GI tract, leading to reduced bioavailability. Moreover, alterations in the conformation of the insulin polypeptide chain structure may lead to protein inactivation and a consequent reduction in its biological activity [7]. While the oral formulation of semaglutide, a peptide drug and GLP-1 receptor agonist for diabetes management, has successfully entered the market, its low bioavailability can result in significant variability in plasma concentrations. However, this challenge is effectively managed through once-daily dosing and a long half-life, which help stabilize steady-state levels and ensure consistent therapeutic effects for patients [9]. In contrast, insulin dosing is highly personalized, requiring precise and dynamic adjustments to maintain optimal blood glucose control and prevent hypoglycemic events. This complexity necessitates additional strategies to achieve effective management, underscoring the differences in treatment approaches between semaglutide and insulin. Various pharmacological approaches have been investigated to address these challenges and enhance the administration and absorption of orally delivered insulin.

This review comprehensively analyzes the recent data from relevant preclinical studies and clinical trials of therapeutic approaches that utilize oral insulin, including their effectiveness and safety profile.

## Insulin formulations

### Nanoformulations

#### Liposomal nanoparticles

Liposomal nanoparticles, composed of a phospholipid bilayer, are nanodrug delivery systems that, when administered orally, can improve the absorption of protein drugs such as insulin. They also have the potential to reduce enzymatic breakdown and immune response activation, while being compatible with living organisms [10]. Two types of liposomal nanoparticles have been documented: hepatic-directed vesicle insulin (HDV-I) and distearylphosphatidylethanolamine-PEG4300-folic acid (DSPE-PEG3400-FA) liposomes. HDV-I is an innovative experimental delivery system comprising insulin and a hepatocyte-targeting molecule embedded in a phospholipid bilayer [11]. In this formulation, the entire amount of insulin is loaded into the HDV to prevent its breakdown by proteolytic enzymes in the upper GI tract, thus enhancing insulin absorption. In addition, hepatocyte-targeting molecule enable the replication of normal physiological insulin administration by specifically targeting hepatocytes. The DSPE-PEG3400-FA liposome formulation incorporates polyethylene glycol (PEG) molecules and folic acid ligands to enhance the stability of the nanoliposomes, hinder mucus penetration, and reduce cellular absorption [12]. Hydrogenated soy phosphatidylcholine was used as a thermally resistant phospholipid in the liposomes, to enhance their durability within the GI system.

Currently, there is a lack of studies examining the effect of oral HDV-I on animals. Researchers have developed a 5-unit HDV-I size two capsule. The stability of this solid oral dosage form has been assessed at temperatures of 5 °C, 25 °C, and 40 °C for five months under low pH conditions, and in blood. Furthermore, the small size of this formulation, with diameter lower than 150 nm, has been shown to render it resistant to enzymatic destruction [11, 13]. In a study by Yazdi, et al. [12] conducted to investigate the antidiabetic effects of DSPE-PEG3400-FA liposomes. The diabetic rat group receiving SC insulin, showed the most significant decrease in blood glucose levels within the initial 2 h, with a peak occurring 1–2 h after injection. Administration of mPEG2000-DSPE-liposome-folic acid, on the other hand, resulted in a greater reduction in glucose levels in the diabetic rats than in the rats receiving subcutaneous insulin, as observed explicitly at the 4 h mark after administration of the formulation. With respect to the pharmacokinetic characteristics, the concentration of insulin in the bloodstream of rats peaked 1–2 h after SC injection. In contrast, 1% and 2% mPEG2000-DSPE-liposome-folic acid achieved their highest levels at 4 h after oral administration [12].

#### Metallic nanoparticles

Metallic nanoparticles consist of a metal core typically enveloped by a shell of an organic material, an inorganic material, or a metal oxide. This review discusses two types of metallic nanoparticles: gold and selenium. Gold nanoparticles are biocompatible, non-toxic, and have a high affinity for many biomolecules, including insulin- and protein-based structures [14, 15]. Selenium (Se), a trace element nutrient, can inhibit the development of metabolic disorders, such as T2DM, by lowering the oxidative stress [16]. There is a scarcity of research on the use of Se nanoparticles for the delivery of oral insulin to produce a hypoglycemic effect equivalent to that of conventional SC administration of insulin mimetics [17].

In 2005, a study examined the use of gold nanoparticles for the delivery of oral insulin. Two types of nanoparticles were orally administered to diabetic Wistar rats: Au-Ins and Au-Asp-Ins, in which insulin was loaded through covalent linkages and hydrogen bonding, respectively. Rats that received Au-Asp-Ins experienced a maximum decrease in blood glucose levels of 31% at 3 h after delivery, while those that received Au-Ins experienced a 19% decrease. The effectiveness of the Au-Asp-Ins formulation was lower than that of the traditional SC insulin (at a dosage of 5 IU/kg), where the SC insulin demonstrated significant decrease of 53% in blood glucose levels in diabetic rats, within 2 h after delivery. The Au-Asp-Ins formulation released insulin more rapidly than Au-Ins, which could be attributed to the lower strength of the hydrogen bonding [18].

Insulin-loaded selenium nanoparticles (INS-SeNPs) were formulated using the ionic cross-linking/in situ reduction method. The compound sodium selenite (Na_2_SeO_3_) was introduced to a complex formed by insulin and chitosan. This was followed by the addition of reduced L-glutathione (GSH), which caused Se^4+^ to convert to Se at its exact location, creating a solid substance. Upon oral administration of three different doses of INS-SeNPs (12.5, 25, or 50 IU/kg) to normal Sprague–Dawley and T2DM Goto-Kakizaki rats, the dosage of 50 IU/kg had a significant hypoglycemic effect in normal rats, similar to that of SC insulin (1 IU/kg). This demonstrates that INS-SeNPs can resist degradation by digestive enzymes and enhance insulin absorption. The significant 21% reduction in blood glucose levels in rats treated with blank SeNPs, compared to ~ 20—45 of INS-SeNPs with insulin dose of 12.5, 25, or 50 IU/kg, demonstrated the antidiabetic properties of Se, which also contributes to this effect. In diabetic rats, the hypoglycemic effect of INS-SeNPs was dose-dependent, with a significant and long-lasting effect observed at the lowest dosage of 12.5 IU/kg. The pharmacological bioavailability showed a reduction as the dose increased in the order 12.5, 25, and 50 IU/kg, with values of 9.15%, 6.75%, and 4.12%, respectively. Based on the daily dosage in rat studies, it is estimated that the equivalent Se intake from INS-SeNPs in humans is 2 mg/day. No toxic effects are believed to be associated with Se in the human body, and Se has not been reported to affect the human body, either genetically or immunologically [19].

The summary of the metallic nanoparticle formulation is demonstrated in Table 1.

**Table 1:** Preclinical studies on metallic nanoparticle formulation of oral insulin

Metallic nanoparticle formulation	Effect on blood glucose levels in animals	Relative bioavailability (%)	Safety profile	Reference
Au-Ins	19% maximal reduction	Not available	Not available	[18]
Au-Asp-Ins	31% maximal reduction	Not available	Not available	[18]
INS-SeNPs	Significant, long-acting, and dose-dependent hypoglycemic effect, proportionate to dosage	4.12–9.15	No possible toxic effects	[19]

#### Polymeric nanoparticles

Polymeric nanoparticles are small particles capable of encapsulating active substances, either by trapping them within the polymeric core or absorbing them onto their surface. Many of these nanoparticles consist of biopolymers, such as albumin, collagen, gelatin, keratin, and silk proteins; or polysaccharides, such as alginate and chitosan [20]. These biopolymers are biocompatible, making them both biodegradable and non-toxic, even after prolonged exposure. The polymeric nanoparticles mentioned below have been classified into single-stage or multi-stage formulations based on the level of complexity of their preparation.

##### Single-stage polymeric nanoparticles

Chitosan is a biocompatible polymer frequently used for drug delivery. A study conducted by Sonaje, et al. [21] utilized pH-responsive nanoparticles composed of chitosan and poly(γ-glutamic acid) to administer insulin aspart (a rapid acting insulin), orally. A transepithelial electrical resistance test subsequently carried out on Caco-2 cells showed that the cationic chitosan nanoparticles temporarily broke the strong bonds between the cells. Another study examined the pharmacodynamics of oral insulin aspart, SC insulin aspart, normal insulin, neutral protamine Hagedorn (NPH) insulin (an intermediate-acting insulin), and insulin detemir (a long-acting insulin). The pharmacokinetic investigation involved a comparison of SC administration of insulin aspart and NPH insulin. In a rat model of diabetes, SC administration of insulin aspart or normal insulin resulted in a pronounced reduction in blood glucose levels over a comparatively shorter period of time (2–3 h). Conversely, when insulin aspart-loaded nanoparticles were administered orally, there was a noticeable but slower decrease in blood glucose levels within 6 h of delivery compared to SC insulin, followed by a gradual increase. Insulin aspart exhibited enhanced absorption kinetics when administered by the SC route, with the maximum plasma concentration (C_max_) achieved 30 min following oral administration, as compared to 3 h post-injection by the oral route. Oral administration of insulin aspart reduces the likelihood of hyperinsulinemia, a condition characterized by excessive insulin levels, as it is absorbed gradually and continuously over an extended duration, without causing rapid spikes. Oral insulin aspart has the potential to be used instead of basal insulin therapy based on its pharmacokinetic profile, which is similar to that of subcutaneous NPH insulin. The nanoparticles used to deliver oral insulin aspart had a relative bioavailability of 15% [21].

Thiolated chitosan nanoparticles (TCNPs) loaded with insulin are another type of polymeric nanoparticles based on chitosan that are currently under investigation. Extended insulin release from TCNPs has been reported in vitro, at pH 5.3. When diabetic rats treated with streptozotocin were orally administered insulin-loaded TCNPs (Ins-TCNPs), their blood glucose levels decreased more slowly and the amount of insulin in their blood increased compared to when insulin was injected via the SC route. The extended duration of insulin action is likely attributable to the interaction between the thiol group and glycoproteins in the intestinal mucus. Assessment of the biocompatibility of TCNPs in Caco-2 cells revealed that the cell viability remained unchanged at TCNP concentrations below 1000 μg/mL, indicating no substantial impact. However, the viability of the cells steadily declined as the concentration of TCNPs rose above 1000 μg/mL [22].

The use of trimethylchitosan (TMC) nanoparticles to orally deliver insulin was investigated by conjugating them to glycyl-glycine (GG) and alanyl-alanine (AA). Previous studies have indicated that conjugating polymers with dipeptides can improve their absorption across enterocytes by allowing them to pass through the intestinal layer using the proton-coupled oligopeptide transporters PepT1 and/or PepT2. Characterization of the nanoparticles for subsequent experiments determined that a concentration of 1.5 mg/mL for both polymer conjugates and insulin concentration was preferred for the particle fabrication. An experiment conducted in rats with diabetes demonstrated that oral administration of trimethylchitosan-carboxymethyl-glycyl-glycine (TMC-CM-GG) and -alanyl-alanine (TMC-CM-AA) nanoparticles reduced the concentration of glucose in the blood over a period of 8 h after delivery. The decrease in serum glucose concentration following the oral administration of TMC-CM-GG was similar to that observed after SC insulin administration, with no significant difference. Specifically, the blood glucose level was reduced by 46.8% of the initial level at 8 h after oral administration, compared to the observed reduction of 64.4% at 2 h after SC administration. Conversely, ingestion of TMC-CM-AA nanoparticles resulted in a reduction of 54.9% in the concentration of glucose in the bloodstream at 8 h, as compared to ingestion of insulin solution, which showed no effect. The serum insulin levels attained by dipeptide conjugation were approximately half of those attained by SC insulin injection. The relative bioavailability was 17.19% for TMC-CM-GG and 15.46% for TMC-CM-AA. In the toxicity tests, the addition of all polymer conjugates to Caco-2 cells led to survival rates of > 65% and > 50% at concentration of 1 and 5 mg/mL, respectively. In comparison, the controls, which are insulin solutions with concentration of 1 and 5 mg/mL, exhibited survival rates of 87.3% and 76.1%, respectively [23].

Furthermore, researchers are examining the potential of poly(isobutylcyanoacrylate) as a polymeric drug carrier owing to its stability and ability to break down naturally in the human body. When transformed into nanocapsules, their small size (< 300 nm in diameter) allows for absorption in the intestines [24, 25]. Thus, it was hypothesized that the use of poly(isobutylcyanoacrylate) nanocapsules enhances the assimilation of insulin. Damgé, et al. [26] showed that poly(isobutylcyanoacrylate) nanocapsules effectively lowered blood glucose levels in streptozotocin-induced diabetic fasting rats, within 2 d of oral administration. Hence, a more recent investigation examined the extent to which insulin could be absorbed into the bloodstream following the ingestion of poly(isobutylcyanoacrylate) nanocapsules. The nanocapsules were created by means of interfacial polymerization using isobutylcyanoacrylate. These findings demonstrated that oral administration of insulin-loaded nanocapsules at the dose of 50 mIU/kg effectively transported insulin into the systemic circulation, as evidenced by a significant increase in blood insulin levels in the diabetic rats. The measured plasma insulin levels ranged from 50 to 240 mIU/L. The insulin concentration in the blood of the rats varied, with the rats falling into two distinct categories: those with high absorption and those with low absorption. Although the insulin level increased, there was no detectable drop in blood glucose levels even 2 d after the delivery of the nanocapsules, which contradicts the findings of previous studies. When insulin was administered to rats through injection at a dose of 5 IU/kg, blood insulin levels increased to 5500 mIU/L in normal rats and 4000 mIU/L in diabetic rats. However, the normal rats showed a greater decrease in blood sugar levels than the diabetic rats. This suggests that the lack of hypoglycemic effect in rats treated with orally delivered insulin-loaded nanocapsules may be due to insulin resistance. Insulin resistance in rats requires high insulin concentrations in the bloodstream to exert an impact [27].

The hypoglycemic effects of insulin-containing Eudragit RS-100 (ERS-100) nanoparticles have been studied in diabetic rabbits and sheep. According to Olya, et al. [28] and Trapani, et al. [29], when taken orally, the polymeric system ERS-100 shields the peptides it contains from denaturation. ERS-100 does not dissolve at physiological pH but swells when added to water, which prevents enzymes from breaking it down in enteric formulations [30]. In vivo studies using diabetic rabbits found that gavage administration of ERS-100 nanoparticles containing insulin (ILNP) significantly reduced blood glucose levels by 40 mg/dL and maintained this reduction for 2 d. Furthermore, a study in sheep treated with oral ILNP showed that the blood glucose levels on Day 5 were significantly lower than those in the control group. It was also noted that post-treatment insulin levels were not affected at any sampling time, suggesting that the pancreas might have secreted insulin through a negative feedback mechanism upon oral administration of ILNP. Cortisol levels, which are known to be associated with gluconeogenesis, were also significantly reduced in the ILNP-treated group, as compared to that in the controls [28].

ERS-100 nanoparticles have also been mixed with poly(ε-caprolactone), a biodegradable polymer, to make insulin nanoparticles using the double emulsion method. In an in vivo study conducted to evaluate the effects on blood glucose levels in diabetic rats, as compared to the control group, nanoparticles encapsulating 100 IU/kg insulin significantly reduced glycemia by 41% at the 4th hour post-oral administration, and this effect lasted at least 8 h. Similarly, the area under the receiver operating characteristic curve (AUC) of blood glucose levels reduced by 23% and 38% upon treatment with nanoparticles containing 50 and 100 IU/kg insulin, respectively, as compared to that in the control group. The relative bioavailability of the insulin-loaded nanoparticles was 13.21% [31].

In case of PEGylated starch acetate nanoparticles, amphiphilic PEG with a molecular weight of 1900 Da was conjugated with hydrophobic starch acetate by means of spontaneous aggregation to form micelles with hydrophobic cores. An in vitro study of its insulin release profile showed that ~ 20% and ~ 55% of insulin was released at pH 1.2 and 7.4, respectively, within 2 h. On the other hand, after 8 h in simulated intestinal fluid with a pH of 6.8 and phosphate-buffered saline with a pH of 7.4, 60% and 80% of insulin was released, respectively. In contrast, the observed cumulative insulin release of 20% after 2 h in the simulated gastric fluid was relatively low, considering that this was the average gastric transit time. The sustained release of insulin from the nanoparticles observed at neutral or basic pH was achieved by diffusion or swelling of the nanoparticles, which stabilized the insulin by preventing it from self-association upon release from the nanoparticles. Assessment of the cytotoxicity of PEGylated starch acetate nanoparticles found that > 98% of the L929 cells incubated with the nanoparticles were viable. The nanoparticle-induced hemolysis was also insignificant, as compared to that induced by toxic PEG nanoparticles with low molecular weights (500 and 800), thereby demonstrating its safety as a delivery system [32].

A starch-based nanocomposite consisting of short-chain glucans (SCGs), which are debranched starches, has been investigated for its potential as an insulin delivery nanocarrier. Proanthocyanidins (PACs) were added to stabilize insulin and facilitate hydrogen bonding between SCG, insulin, and PAC. After oral administration of insulin-SCG-PAC to diabetic rats, the blood glucose levels decreased by up to 36.84% at the 3rd hour and were sustained for the entire study period of 8 h, in contrast to that in case of the SC insulin injection, where the blood glucose levels spiked back to its initial level at 6 h. This may also be explained by the fact that smaller particle size results in better insulin absorption in the intestine. The pharmacological activity of insulin-SCG-PAC was 6.98%, while that of the oral insulin solution was 0.64%. A cell viability test conducted using undifferentiated Caco-2 cells to investigate the cytotoxicity of the nanoparticles showed that the insulin-SCG-PAC nanocomposite was low in cytotoxicity, with relative cell viability above 90% at concentrations of 125 to 500 μg/mL and even > 85% at the higher concentration tested, which was 1000 μg/mL [33].

The preclinical studies on single-stage nanoparticle formulations are summarized in Table 2**.**

**Table 2:** Preclinical studies on single-stage polymeric nanoparticle formulations of oral insulin

Single-stage polymeric nanoparticle formulation	Effect on the blood glucose levels in animals	Relative bioavailability (%)	Safety profile	Reference
Chitosan & γ-PGA	Significant and gradual reduction in blood glucose levels within 6 h	15	Not available	[21]
TCNPs	Prolonged reduction in blood glucose levels	Not available	No significant change in cell viability below the concentration of 1000 μg/mL	[22]
TMC-CM-GG nanoparticles	Reduction to 46.8% of initial level at 8th hour (No significant difference to that seen in case of SC insulin)	17.19	Cell viability > 65% and > 50% at concentrations of 1 and 5 mg/mL, respectively	[23]
TMC-CM-AA nanoparticles	Reduction to 54.9% of initial level at 8th hour	15.46	Cell viability > 65% and > 50% at concentrations of 1 and 5 mg/mL, respectively	[23]
Poly(isobutylcyanoacrylate) nanocapsules	No decrease in blood glucose levels	Not available	Not available	[27]
ERS-100 nanoparticles	Significant reduction by 40 mg/dL, maintained for 2 d	Not available	Not available	[28]
ERS-100 + poly(ε-caprolactone) nanoparticles	Reduction by 41%, which was maintained for at least 8 h	13.21	Not available	[31]
PEGylated starch acetate nanoparticles	Not available	Not available	Cell viability > 98%	[32]
SCG-PAC nanocomposite	Reduction of 36.84% in blood glucose levels, which was maintained for at least 8 h	6.98	Cell viability > 90% and > 85% at concentrations of 500 and 1000 μg/mL, respectively	[33]

##### Glucose-responsive polymeric nanoparticles

Hypoglycemia is a well-known adverse effect of insulin therapy, with symptoms ranging from confusion and anxiety to potential loss of consciousness and even death. Research has explored methods to automate insulin delivery based on blood glucose levels, including glucose-sensitive subcutaneous insulin, which shows promise in reducing the incidence of hypoglycemic episodes. This approach involves the binding of glucose to a macrocycle linked to insulin, enabling the controlled release of insulin in response to rising glucose levels [34]. Similarly, in the context of oral administration, glucose sensitivity can be achieved through redox reactions of specific excipients under hyperglycemic conditions, which modulate the release of insulin accordingly. Zhou, et al. [35] designed a glucose-responsive delivery system that releases insulin depending on the blood glucose concentration. This was achieved by having functional sections like phenylboronic acid (PBA), glucose binding proteins and glucose oxidase (GOx), incorporated in their particles as well as designing a 2-nitroimidazole-l-cysteine-alginate (NI-CYS-ALG) polymer, where each functional group plays an important role for the delivery: (1) *2-Nitroimdazole (NI):* Under hyperglycemic conditions, glucose oxidase (GOx) loaded into the particles alongside insulin, induces hypoxic conditions leading to the reduction of NI to the hydrophilic 2-aminoimidazole. (2) *L-Cysteine*: The thiol groups interact with the cysteine-rich subdomains of mucus glycoproteins in the small intestine and therefore prolongs the resistance time within the GI tract and increases the pH stability of the system. Additionally, it improves intestinal permeability as it changes the distribution of F-actin and ZO-1. (3) *Alginate*: the hydrophilic polyanion polymer has mucoadhesive properties. When incubating the particles with different concentrations of glucose, the size gradually increased from 230 to 616 nm in the first hour and to 977 nm within 6 h. This size increase was due to the reduction of NI in the hyperglycemic condition caused by GOx. Furthermore, in hyperglycemic solution (400 mg/dL glucose), the particles had a high insulin release ratio (30.43 ± 15.64% in the first hour and 87.56 ± 6.95% after 10 h), due to the dissociation of the particles. Particles exposed to normal conditions (100 mg/dL glucose), only released part of the insulin (25.10% ± 5.97% within 10 h) [35].

Upon administration to diabetic rats, both formulations, one containing co-loaded insulin and GOx, and the other with insulin alone, exhibited a strong hypoglycemic effect. Subcutaneous injection of insulin decreased the blood glucose level to 12.2 ± 1.0% after 1 h, however the levels returned to hyperglycemia after 4 h. The insulin-loaded particles managed to reduce the levels to 45.1 ± 4.1% of the initial concentration by 8 h and kept these levels for another 6 h. Particles loaded with GOx and insulin managed to decrease blood glucose levels to 30.2 ± 1.9% for 2 h and could maintain euglycemic levels up to 12 h, show-casing the successful design of a glucose-responsive drug delivery system able to reduce and keep blood glucose to euglycemic levels [35].

##### Multi-stage polymeric nanoparticles

**Fig. 3:**
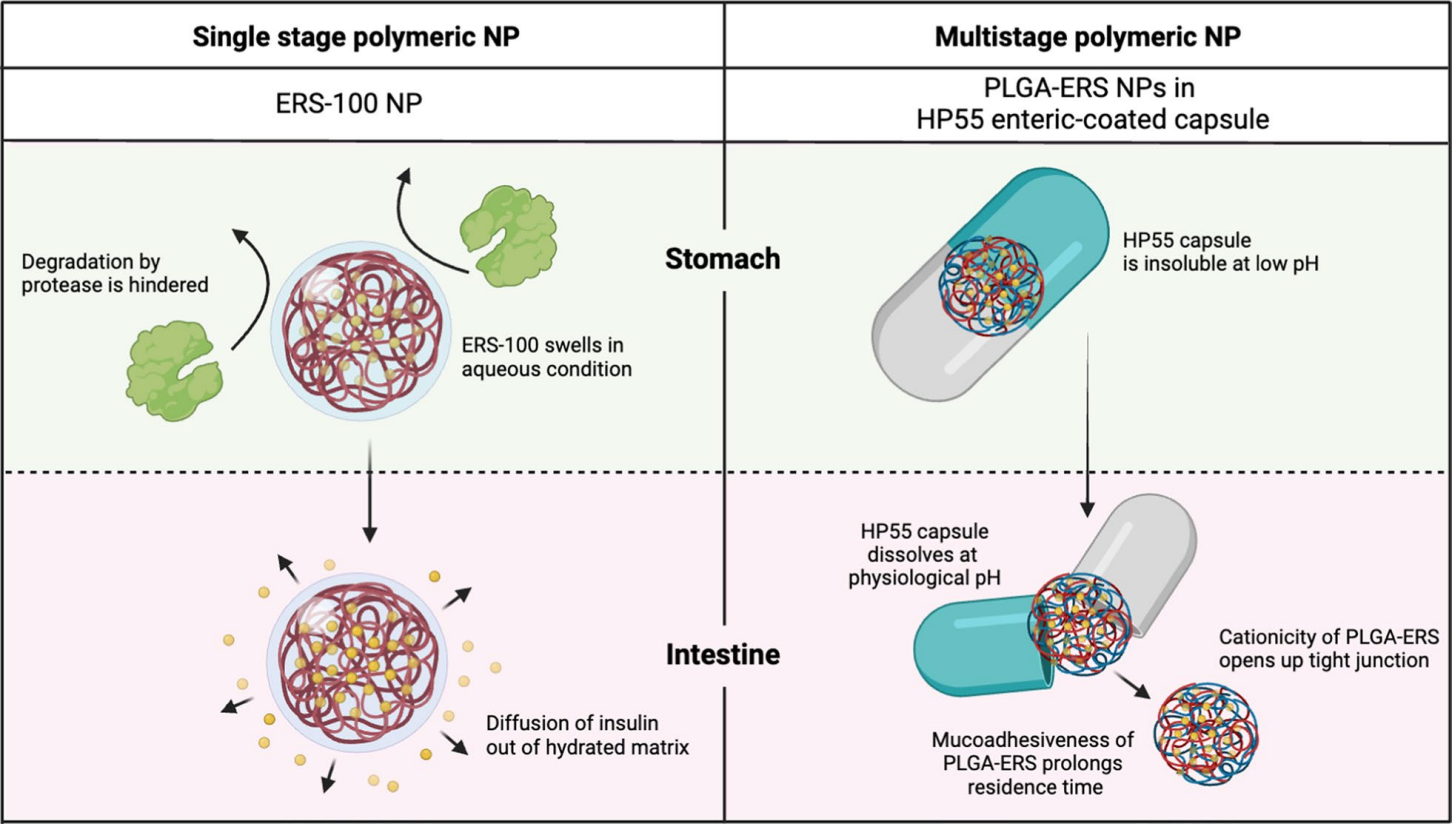
Comparison between single-stage and multi-stage polymeric nanoparticles. Comparison between single-stage and multi-stage polymeric nanoparticles. ERS-100 NPs and PLGA-ERS NPs in HP55 enteric-coated capsules were compared on the basis of ERS-100 as the common excipient. Adapted from Biorender.com

In this study, a two-stage polymeric nanoparticle delivery system was developed. The first stage is pH-sensitive, while the second involves sticking to the mucosa. Collectively, these stages can overcome the barriers that make oral insulin absorption challenging [36]. First, the outer layer of the hard gelatin capsules was covered with hydroxypropyl methylcellulose phthalate (HP55), an enteric polymer sensitive to pH, with a pK_a_ of 5.50. This prevents oral insulin from losing its efficacy due to enzyme breakdown and the low-pH environment of the stomach. Unlike ERS-100, HP55 dissolved in the upper part of the small intestine when the pH was higher than its pK_a_. This allowed the nanoparticles to escape from the capsule that breaks apart. Second, cationic nanoparticles containing insulin made of poly(lactic-co-glycolic acid) (PLGA) and ERS were absorbed across the intestine. Despite being non-biodegradable, ERS-100 is biocompatible and can adhere to the mucosal layer of the GI tract [29]. The enhanced absorption is possibly due to the longer residence time in the intestine and its cationicity, which opens tight junctions. The insulin-loaded PLGA/ERS nanoparticles were formulated by means of ultrasonic emulsification using the multiple-emulsion solvent evaporation method. In vitro results showed that HP55-coated capsules reduced insulin release at pH 1.2 from 90 to 10%. However, PLGA/ERS nanoparticles released insulin at pH 7.40 in the same way with or without the HP55-coated capsule, suggesting that the enteric coating does not change insulin release at this pH, as demonstrated in Fig. 3 with comparison to ERS-100 nanoparticles, single-stage nanoparticles. An in vivo study on diabetic rats showed that oral administration of enteric-coated capsules filled with insulin-loaded PLGA/ERS nanoparticles induced a hypoglycemic effect of 32.9% (defined as the area above the curve of the plasma glucose level), at a dose of 50 IU/kg, with a time to reach peak concentration (T_max_) of 10 h. This was comparable to the results obtained in rats injected with SC insulin at a dosage of 5 IU/kg (35.20%), with T_max_ at the 2nd hour. The delivery system demonstrated a prolonged hypoglycemic effect in a diabetic rat model, with a pharmacological availability of 9.2%. These results suggested that the two-stage delivery system is a potential approach for improving the efficacy of oral insulin delivery [36].

**Fig. 4:**
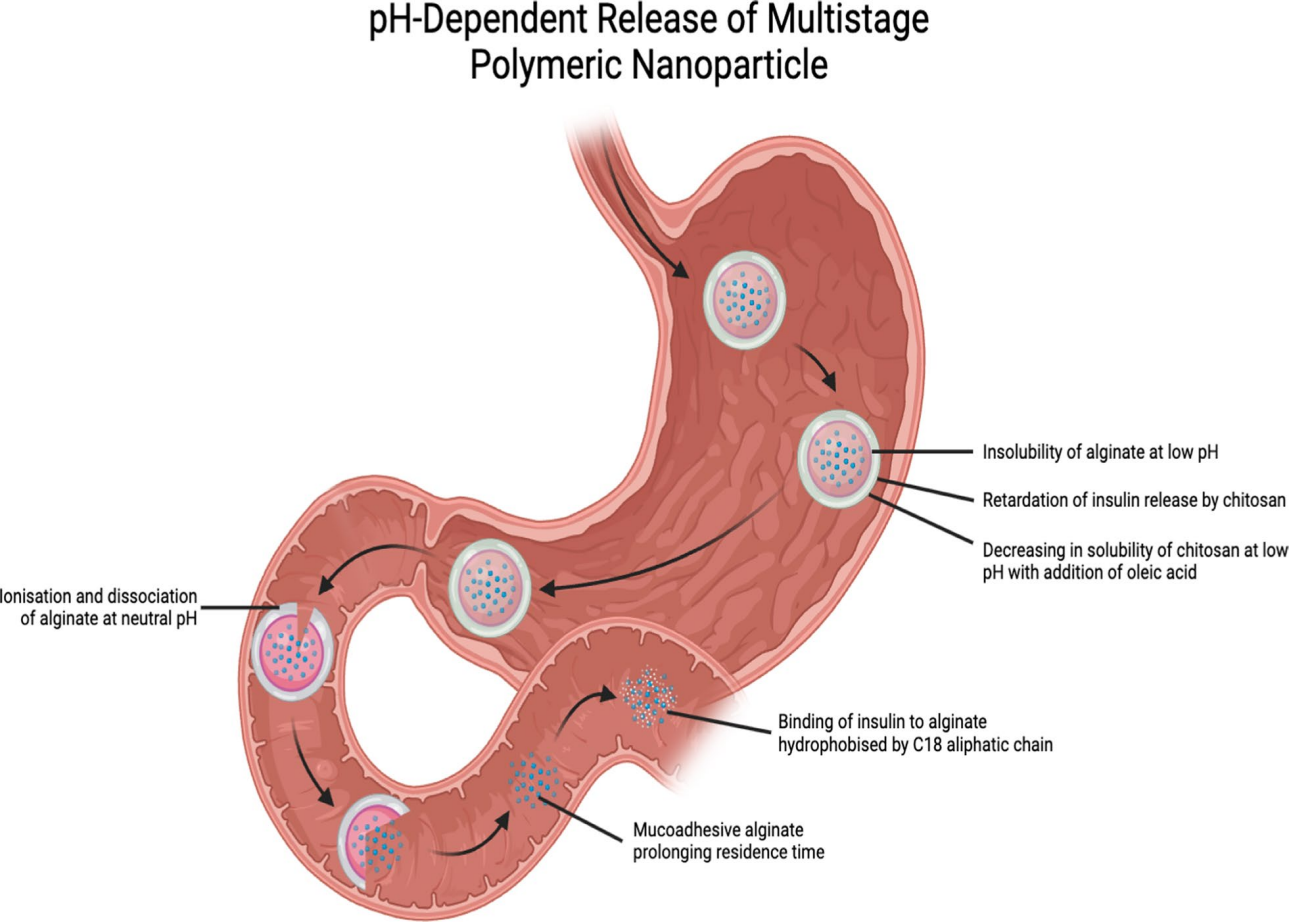
pH-dependent release of chitosan-oleic acid conjugate-coated calcium alginate beads (CCAB) loaded with alginate-C18 nanoparticles (AC18N). Adapted from Biorender.com

Nanoparticles in beads have also been investigated, especially insulin-loaded alginate nanoparticles microencapsulated in pH-sensitive alginate beads and covered with a layer of chitosan-oleic acid. Alginate beads possess protective properties that delay the release of nanoparticles into the gastric environment. The mucoadhesive properties exhibited by alginate following dissociation and ionization at neutral pH in the intestines also prolong the residence time of the nanoparticles in the intestines. In addition, alginate can bind to dietary glucose in the intestines and impede its absorption into the bloodstream. Adding oleic acid to the chitosan backbone slowed the release of the payload. This is because chitosan does not dissolve easily under acidic conditions, which keeps its interactions with alginate intact. In the drug release profile and in vivo studies, alginate-C18 nanoparticles (AC18N) were used. The addition of C-18 aliphatic chains can make alginate more hydrophobic, preventing it from interacting with insulin and blocking its release. Chitosan-oleic acid conjugate-coated calcium alginate beads (CCAB) loaded with AC18N released 2.8% of the insulin loaded in the first 2 h, in simulated gastric fluid at pH 1 (Fig. 4). After moving to simulated intestinal fluid with a pH of 6.8, 24.7% of the loaded insulin was released over the next 4 h. The AC18N-CCAB also caused a greater decrease in the blood glucose levels in the diabetic rats than that observed in the control groups and upon treatment with SC insulin and free insulin-loaded nanoparticles. However, an exception was observed in the case of the sample obtained at 30 min, possibly due to the digestion of the polysaccharide content of the formulation. This was consistent with the blood insulin level at 24 h, which was significantly higher in the AC18N-CCAB-treated diabetic rats than that observed in the case of treatment with insulin-loaded AC18N. It is worth mentioning that CCAB loaded with insulin-free AC18N induced a greater reduction in blood glucose levels than that loaded with insulin-containing AC18N, owing to the sugar-binding property of alginate. Upon assessment of the toxicity profile in HT29 cells, insulin-loaded AC18N was comparable to the control, indicating low toxicity of AC18N [37].

**Fig. 5:**
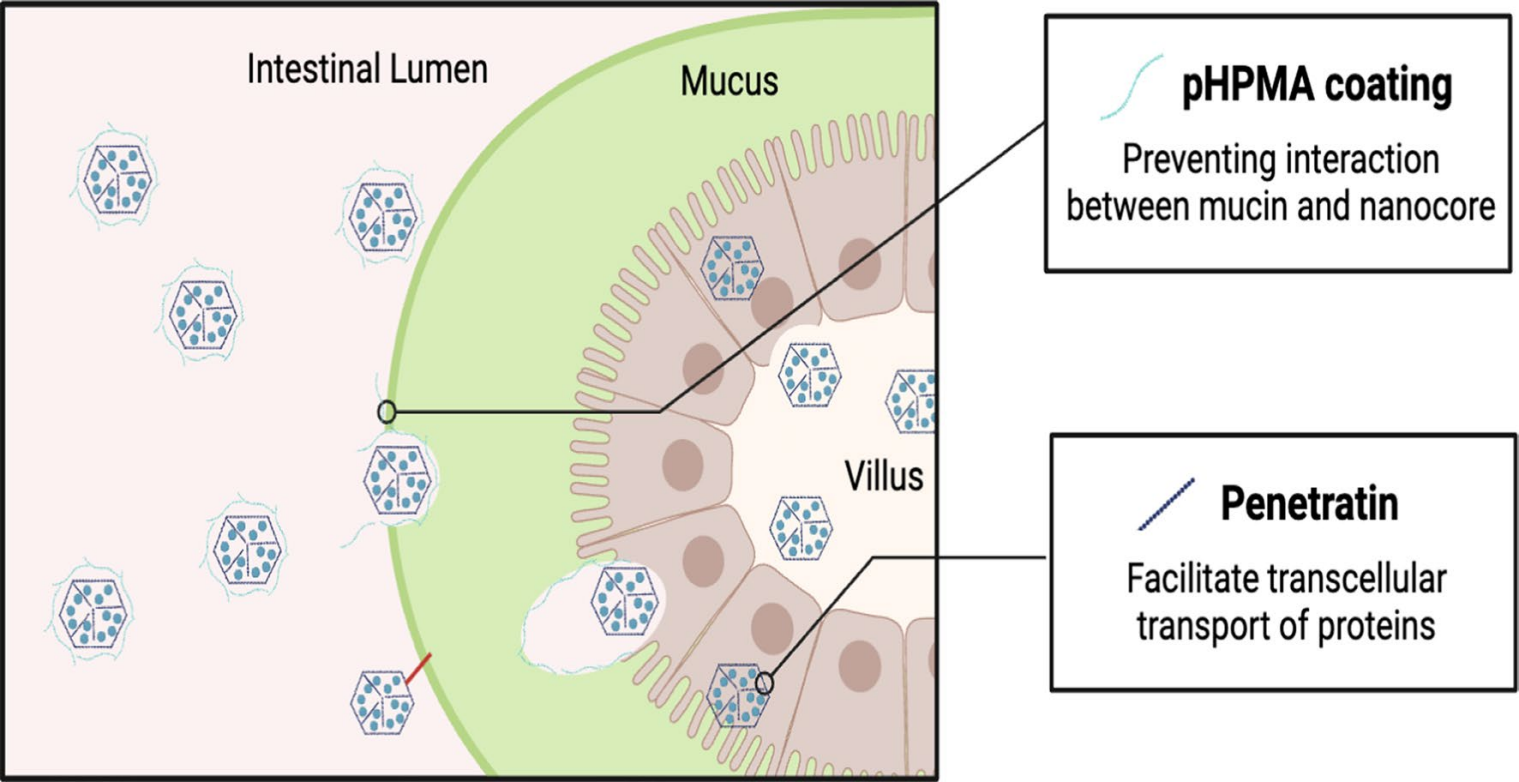
Enhanced permeation of self-assembled N-(2-hydroxypropyl) methacrylamide (pHPMA) polymeric nanoparticle across intestinal lining. Interaction between the nanocore uncoated with pHPMA and the mucin is depicted in red line which hinders the insulin from passing through the mucus barrier. Adapted from Biorender.com

Another potential insulin carrier are self-assembled N-(2-hydroxypropyl) methacrylamide (pHPMA) polymeric nanoparticles. The nanocomplex (NC) core consists of anionic insulin mixed with polycationic penetratin, a cell-penetrating peptide, while coating layer consists of dissociable negatively charged hydrophilic pHPMA that self-assembles upon the addition of the NC to the pHPMA solution. pHPMA with varying MA-GG-OH monomer ratios has been used to determine the molecular structure. Using an Ussing Chamber System to simulate the mucus layer, epithelial cells beneath the porcine intestinal mucus, and a semipermeable membrane, it was found that the pHPMA coating was able to improve permeation, as compared to that observed in case of uncoated NCs, because of its ability to curtail the interactions between the NCs and anionic/hydrophobic regions of the mucin structure in the mucus (Fig. 5). However, it became less permeable as the charge density of pHPMA increased, probably due to the repulsion between pHPMA and mucus, which are both negatively charged. The ability of pHPMA-coated NCs to permeate through the mucus was further demonstrated in terms of the Brownian movements observed in the mucus and an in vitro insulin uptake study, whereas uncoated NCs were trapped in the mucus owing to interactions. In an in vivo study comparing the blood glucose levels-lowering effects of saline, PO-free insulin solution, SC insulin solution, uncoated NCs, and pHPMA nanoparticles with the lowest charge density in diabetic rats, pHPMA nanoparticles demonstrated a maximal reduction of 50% in blood glucose levels, at 4 h post-administration with a dosage of 75 IU/kg. Orally administered free insulin had virtually no effect on glucose levels, whereas the NCs caused a slight decrease. SC insulin showed the greatest reduction, which peaked at 2 h post-administration; this was reflected in the serum insulin level as well, which peaked at the first hour. Serum insulin levels in diabetic rats administered pHPMA nanoparticles peaked at the 4th hour. The pharmacological availability of the pHPMA nanoparticles was 6.61%, more than 2 times that of the uncoated NCs (2.60%), validating the function of the pHPMA coating. In vitro safety evaluation using the HT29-MTX-E12 (E12) cell line demonstrated no significant cytotoxicity over the concentration range of 50 to 200 μg/mL [38].

In a study on the formulation of insulin-loaded alginate/dextran sulfate nanoparticles (ADS-NPs), the nanoparticles were dual-coated with chitosan and albumin (ALB). As an anionic copolymer, dextran sulfate can improve the loading of hydrophilic drugs into alginate. Coating with ALB can prevent the proteolytic degradation of insulin by blocking protease access and stabilizing the nanoparticles in both acidic and intestinal environments. A study on the insulin release profile in simulated gastric and intestinal media showed that dual-coated nanoparticles (ALB-NPs) could retain insulin encapsulated at pH 1.2 and burst-release insulin at pH 5.5. This is possible due to the insolubility of alginate at low pH and cross-linking between the positively charged alginate and dextran sulfate. Additionally, at pH 7.4, a sustained release profile of insulin was observed, as chitosan, which is soluble at a lower pH, became insoluble at this pH and could retain insulin. In an in vitro study of the insulin permeation profile of these nanoparticles with a triple co-culture of the Caco-2/HT29 cells adapted to methotrexate (HT29-MTX) (a human colon cancer cell-line)/Raji B (a lymphoblast-like cell-line) cell model, which mimics the monolayer of the intestine, the permeation of ALB-NPs was found to be significantly higher than that of non-encapsulated insulin. At pH 7.4, the insulin released from the ALB-NPs was almost 100% after 3 h, consistent with the release profile. An in vitro cell viability study using AGS (a human gastric adenocarcinoma cell-line), Caco-2, and HT29-MTX cell lines incubated with different concentrations (0.10, 0.25, 0.50, and 1.00 mg/mL) of ALB-NPs showed that the nanoparticles did not significantly lower the cell viability across all cells, as compared to the negative control of HBSS-HEPES buffer, except for at the lower concentrations of 0.1 and 0.25 mg/mL in AGS cells [39].

A nanoparticle with an identical composition was studied for its hypoglycemic effect upon oral administration. Insulin-loaded nanoparticles were found to achieve sustained hypoglycemic effects in diabetic rats that were comparable between the doses of 50 and 100 IU/kg; the absence of a dose–response effect in this case could be attributed to saturation. The hypoglycemic effect achieved by both concentrations of insulin-loaded nanoparticles was significantly different from that of unencapsulated insulin between the 8th and 12th h. For the biodistribution study in diabetic rats, where technetium-99 m-albumin (^99m^Tc-BSA) was used for radiolabeling the nanoparticles, the radioactivity of the ^99m^Tc-BSA nanoparticles in the stomach wall increased only 60 min after oral administration and then decreased steeply in the stomach contents, and finally subsided to < 50% after 90 min. In the small intestine wall, the radioactivity of ^99m^Tc-BSA nanoparticles between 120 and 180 min was significantly higher than that of the control group, ^99m^Tc-BSA, in which case the radioactivity plummeted at 120 min, showing that retention was associated with the interaction between the dual coating and epithelial cells [40].

The preclinical studies related to multi-stage polymeric nanoparticle formulations are summed up in Table 3.

**Table 3:** Preclinical studies on multi-stage polymeric nanoparticle formulations of oral insulin

Multi-stage polymeric nanoparticle formulation		Effect on the blood glucose levels in animals	Relative bioavailability (%)	Safety profile	Reference
Inner layer	Outer layer				
PLGA-ERS nanoparticles	HP55 enteric-coated capsule	Induced a hypoglycemic effect of 32.9%	9.2	Not available	[36]
AC18N	CCAB	Remarkable reduction, as compared to that seen in case of SC insulin	Not available	No significant difference from control	[37]
Penetratin-containing nanocomplex	pHPMA coating	50% maximal reduction at the 4th hour	6.61	No significant cytotoxicity	[38]
ADS-NPs	Chitosan/albumin dual coating	Non-dose-dependent, significant, and sustained reduction	Not available	No significant decrease in cell viability	[39, 40]

#### Porous silicon nanoparticles

Porous silicon nanoparticles have proven effective as carriers for various drugs and routes of administration. Due to their high loading capacity, customizable surface chemistry, and resilience to harsh conditions, such as those encountered in the gastrointestinal tract, they are ideal candidates for oral drug delivery [41]. Moreover, the restricted spaces within their pores contribute to enhancing drug solubility by preventing the formation of crystalline materials [42].

Rao et al. [43] used these advantages by designing a virus-mimicking porous silicon nanoplatform modified with poly (pyridyl disulfide ethylene phosphate/sulfobetaine) (P(PyEP-g-SB) polymers to improve mucus permeability and cellular internalization. By conjugating dodecyl sulfobetaine (SB) to the side chains of poly (pyridyl disulfide ethylene phosphate) (PPyEP) the generated degradable zwitterionic polyphosphoester resembled viral polymer molecular brushes. The zwitterionic side chains facilitated interaction with intestinal mucus and when the phosphoester moieties start to degrade due to the intestinal alkaline phosphatase (IAP), the backbone units and the cationic cores of the nanoparticles facilitate the cellular uptake [43].

Assessment of cellular uptake of the functionalized particles in Caco-2 cells revealed that positively charged groups increased cellular uptake and with increasing SB units, mucus penetration and transcellular transport improved. When testing this system in streptozotocin-induced diabetic rats, blood glucose level did not reduce after the oral administration of a free insulin solution or empty particles. After the subcutaneous injection of insulin, the blood glucose levels dropped, but recovered after 4 h. However, after administration of P(PyEP-g-SB0.3)20- AmPSiNPs (50 IU kg^−1^), the blood glucose levels were significantly reduced for 8 h (34.5%). The unfunctionalized AmPSiNPs showed less pronounced blood glucose reduction (10.3%), with the functionalized particles exhibiting a 1.38 fold higher blood insulin release within the first hour. However, both groups resulted in stable levels after 2 h, which indicates that insulin can be released gradually from the particles. Even though the hypoglycemic effect of the functionalized particles prolonged for up to 8 h, the mice were euthanized, as the prolonged fasting could affect the hypoglycemia effect. Functionalized particles exhibited the highest relative oral bioavailability with 4.36%, in comparison of 2.09% with free insulin and 3.47% with unfunctionalized particles [43].

#### Quantum dots

Hunt, et al. [44] designed pH- and enzyme sensitive silver sulfide (Ag_2_S) quantum dots (QDs) for the oral delivery of insulin. In previous works, Ag_2_S QDs improved the oral bioavailability of metformin and nicotinamide mononucleotide 100–10.0000-fold [45, 46]. In order to protect the loaded insulin as well as achieve controlled release, the authors designed a random polymerized chitosan and glucose copolymer (CS/GS) around the quantum dot insulin construct, which is highly sensitive to enzymatic hydrolysis, especially by ß-glucosidase [44].

Human duodenal explants were live-imaged to investigate the cellular uptake of QDs in the small intestine. These tests revealed that after 2–4 min, around 70% of the particles were located in the cytoplasm and after 8 min, the formation of endocytic vesicles and exocytosis could be observed. Furthermore, when insulin was loaded into the QD-INS-CS/GS system, the uptake of insulin was increased 40-fold compared to insulin alone. When testing the system in rats, subcutaneous injection of insulin lead to a 30% increase in body weight, compared to no changes when being treated with QD-INS-CS/GS. Furthermore, no changes in serum biochemistry or lipids were observed [44].

The most important finding in this study was the absence of hypoglycemia when the system was tested in mice, rats and baboons. By utilizing a glucosidase-responsive system, this system uses the correlation between the β-glucosidase chemical activity and blood glucose concentration, suggesting that the degradation of CS/GS is dependent on the glucose concentration [44].

### Microparticles

Microparticles, defined as spherical particles with a size between 1 and 1000 μm in diameter, have also been studied for insulin delivery. Chitosan phthalate microspheres have been used because of their pH-sensitive properties; they display low solubility at low pH and are completely soluble at basic pH. Insulin is successfully entrapped in these microparticles using an emulsion crosslinking technique. An in vivo study on streptozotocin-induced diabetic rats showed that these microspheres demonstrated a maximal decrease in blood glucose levels to 51.54% of the initial level at 6 h post-administration, and the glucose-lowering effect remained significant, as compared to that of an orally administered chitosan phthalate-insulin solution, for at least 16 h post-administration. It was also significantly lower than SC insulin from the 6th to 20th hour, as the minimum glucose level of SC insulin was achieved at the 1st hour post-administration and returned to the initial level thereafter. The relative pharmacological efficacy of this formulation was 18.66%, which is approximately four times higher than that of the oral chitosan phthalate-insulin solution (5.75%), while that of SC insulin was 14.82% [47].

Another form of microparticles is a microparticulate solid-in-oil-in-water emulsion, in which the enteric polymer hydroxypropyl methylcellulose phthalate is present in the aqueous phase [48]. A pH-dependent release profile was demonstrated, in which the percentage of insulin released was higher at higher pH values and was further boosted in the presence of lipase.

### Hydrogels

Hydrogels are three-dimensional network structures consisting of polymers that are physically or chemically crosslinked and hydrophilic, which allows them to retain water and swell without dissolving into aqueous environments [49]. The efficacy and safety profiles of different hydrogel formulations, including polymethacrylic acid (PMAA)- and polysaccharide-based hydrogels such as cellulose and chitosan, have been investigated to deliver insulin.

The PMAA hydrogel and its variants have been studied because PMAA can improve the permeability of hydrophilic compounds across epithelial cells and inhibit the enzymatic action of calcium-dependent proteases by binding to calcium ions [50, 51]. A PMAA-chitosan-PEG (PCP) hydrogel with or without thiolation was developed, in which functionalization with a thiol group made the hydrogel mucoadhesive, thereby improving its retention on the mucus layer and diffusion across the layer. In the case of the non-thiolized PCP hydrogel, the hydrogel loaded with insulin was complexed with methyl-β-cyclodextrin, as its hydrophobic nature can enhance the absorption of hydrophilic insulin across intestinal cells and impede its self-association. In the release study, insulin-loaded PCP demonstrated pH-sensitive properties, as only 10% of insulin was released within 2 h at pH 1.2, whereas a high percentage of it was released within 3 h at pH 7.4. An in vivo study in diabetic rats showed that oral administration of complex insulin-loaded PCP reduced glycemia levels by 30% in 2 h, which was further sustained for 6 h. Despite the level increasing thereafter, it was still 10% lower than that in the control group, which was not administered any treatment even after 10 h. The relative pharmacological bioavailability of the insulin-loaded PCP was 1.95. As for the safety of methyl-β-cyclodextrin, its cytotoxicity towards Caco-2 cells was found to be concentration-dependent, as at a concentration of 25 mM it resulted in a cell viability of 0%, but below 10 mM, it was non-cytotoxic [50].

**Fig. 6:**
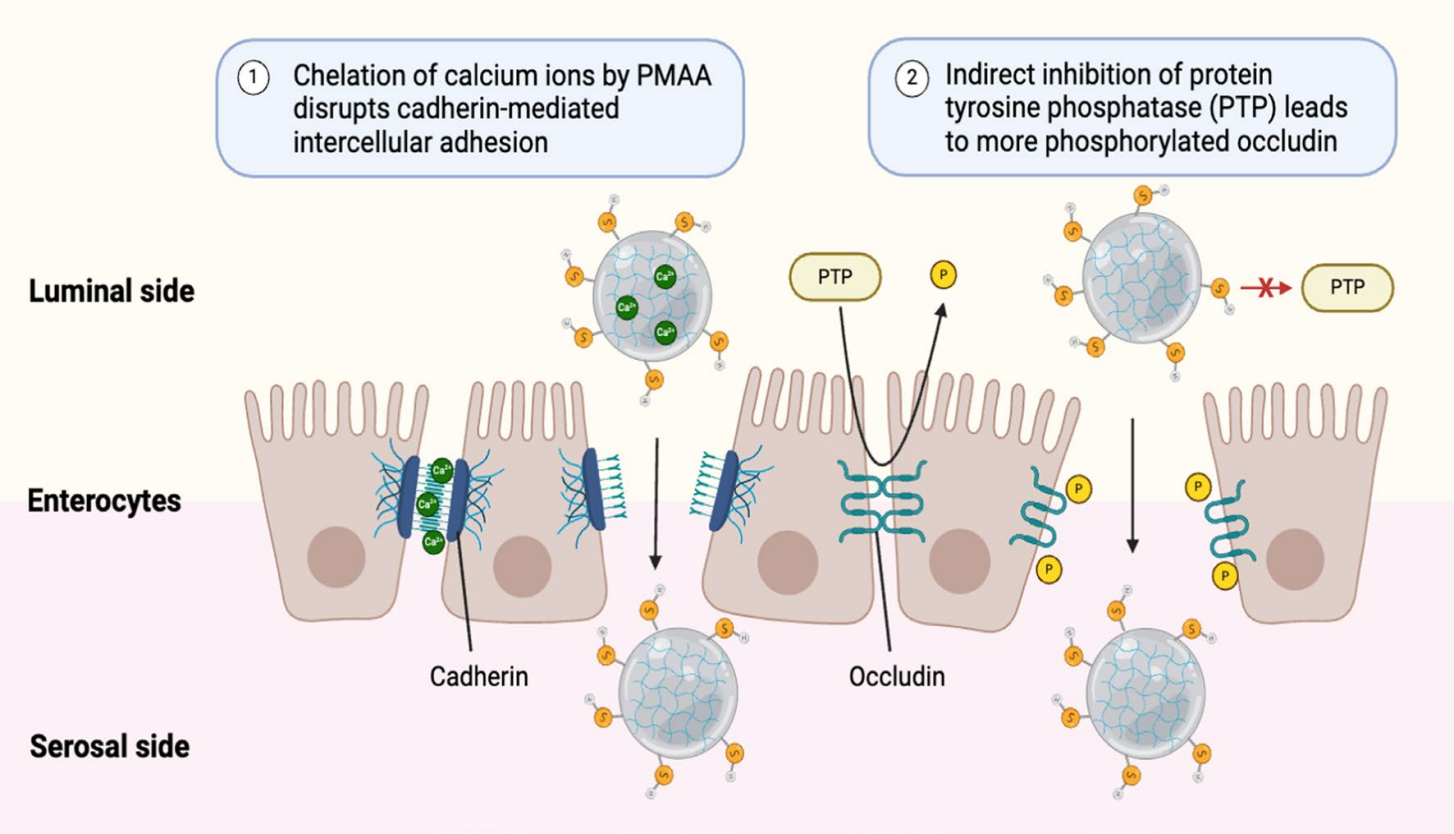
Opening of tight junction through thiolization of PMAA-chitosan-PEG (PCP) hydrogel. Adapted from Biorender.com

In the case of the thiolized variant, thiolization was performed by means of conjugation with cysteine. In Caco-2 cells, insulin encapsulated in cysteine-conjugated PCP microparticles (Cys-PCP) was almost five times more permeable than the unencapsulated control, whereas unconjugated PCP showed three times higher permeability than the control. Both PCP and Cys-PCP caused a > 50% decrease in transepithelial electrical resistance across the monolayer of Caco-2 cells, indicating that tight junctions between cells were loosened as a result of calcium binding and tyrosine phosphatase-mediated inhibition of occluding, a transmembrane protein that is involved in the closing of tight junctions (Fig. 6). PCP and Cys-PCP were 2.5- and 2.8-fold more permeable across rat intestinal tissues than the control, respectively. In diabetic rats, Cys-PCP reduced blood glucose levels by ~ 40% from the initial level in 2 h, and this effect was sustained for over 8 h, whereas PCP reduced them by 15%, which was sustained after 6 h. The relative pharmacological availability of the Cys-PCP particles was found to be 2.45 [51].

PMAA polymers can also be grafted with another polymer, PEG, to form a copolymer hydrogel network known as poly(methacrylic acid-grafted poly(ethylene glycol)) [P(MAA-g-EG)] [52, 53]. As the interactions between both polymers involve hydrogen bonding, the copolymer networks can exhibit pH-sensitive properties, where the hydrogel swells at a higher pH and collapses at a lower pH. P(MAA-g-EG) hydrogel particles encapsulating insulin have been shown to have a favorable hypoglycemic profile in studies on diabetic rats. Administration of 25 IU/kg insulin loaded in the (P(MAA-g-EG)) hydrogel to streptozotocin-induced Wistar diabetic rats resulted in a reduction of ~ 40% in the blood glucose levels for a period longer than 8 h. The bioavailability of insulin ranged from 2.44% to 4.22%, depending on the encapsulated insulin dose and induced diabetic status in the rats, as compared to the range of 0.55% to 0.88% observed in case of the control insulin solution [52]. The size and composition of hydrogel microparticles also affects the oral insulin bioavailability. A study by Morishita, et al. [53] showed that a 1:1 molar ratio of methacrylic acid to ethylene glycol was ideal for inducing the highest percentage of glucose reduction, as indicated by the area above the curve, and had the highest pharmacological availability of 7.4%, as compared to those observed at the ratios of 1:0 and 4:1. With respect to the size, microparticles with diameters of < 53 µm showed the greatest percentage of glucose reduction, highest pharmacological availability, and rapid release of insulin, as compared to microparticles with diameters between 212 and 300 µm [53].

Another example of a copolymeric hydrogel is a hydrogel network composed of succinyl-chitosan-grafted polyacrylamide. While addition of a succinyl group to chitosan improves its hydrophilicity and pH sensitivity, polyacrylamide grafting can increase the number of encapsulated insulin molecules by introducing more amino groups into the hydrogel. The S-chitosan-grafted polyacrylamide (PAA/S-chitosan) hydrogel was found to release 20%–25% insulin under simulated gastric conditions, at pH 1.2, and ~ 98% insulin in the simulated intestinal fluid. This can be attributed to the protonation status of the carboxyl group on S-chitosan at different pH values. At lower pH, the carboxyl group is protonated, causing the hydrogel to shrink, while at higher pH, it is deprotonated and ionized, leading to repulsion between polymers and subsequent release of insulin-loaded polymers. Study of the in vivo efficacy demonstrated induction of hypoglycemic effects in the diabetic mice at 2 h after oral administration, which lasted for at least 6 h. The relative bioavailability of insulin was ~ 4.43%. In terms of in vivo hepatotoxicity, although the levels of the liver enzymes, specifically alanine aminotransferase, aspartate aminotransferase, and lactate dehydrogenase increased as compared to those observed in the control, they were not altered in comparison to the reference values. There was no nephrotoxicity as well, as serum and urine creatinine and urine microprotein levels were all within the reference ranges, despite a significant increase, as compared to that observed in the 0.9% saline control group [54].

β-cyclodextrin is a cone-shaped heptasaccharide with a hydrophobic head and hydrophilic tail that can improve the solubility of chitosan in water. Yang, et al. [55] studied the polymeric hydrogel composed of carboxymethyl chitosan (CMC) grafted with carboxymethyl β-cyclodextrin (CMCD) (CMCD-g-CMC) and found that 92% of insulin was retained in the CMCD-g-CMC hydrogels after 2 h of incubation under simulated gastric conditions, and the percentage released increased to 55% at pH 6.8 and 70% when the pH was further increased to 7.4. Upon testing of the efficacy of this polysaccharide-based hydrogel in vivo, a maximal reduction of blood glucose levels was observed at 6 h after oral administration, which lasted for ~ 12 h, as compared to that observed upon SC injection, which peaked at 2 h, indicating a prolonged effect of the hydrogel, which is ideal to minimize the occurrence of hypoglycemic episodes in diabetic patients. The viability of Caco-2 cells after 24 h of incubation with CMCD-g-CMC hydrogel microparticles with concentrations ranging from 12.5 to 1600 μg/mL was > 96%, and thus, this hydrogel is safe for use in oral drug delivery [55]. These promising results led to a long-term follow-up study of 4 weeks with once daily dosing in diabetic rats, which further showed that insulin-loaded CMCD-g-CMC hydrogels were effective in treating the symptoms of polyphagia, polydipsia, polyuria, and weight loss, as well as in improving fasting blood glucose levels and oral glucose tolerance test outcomes [56].

Besides direct encapsulation of insulin, the hydrogel can also be used as part of the drug delivery system, together with particles loaded with insulin, as seen in the insulin-loaded sodium dodecyl sulfate and β-cyclodextrin (SDS/β-CD) vesicles-chitosan hydrogel. The cationic insulin was entrapped either on the surface of the SDS/β-CD bilayer of the vesicles, inside their cavity, or both, where the vesicles were embedded into the CS hydrogel crosslinked with β-glycerol phosphate, to improve the stability. An in vitro release study revealed that the loaded insulin could be retained in the system at pH 2.5, whereas rapid release was observed at the pH levels of 6.8 and 7.4, with or without enzymes added to the medium, and the percentage of residual insulin after incubation in the simulated intestinal fluid was significantly higher in the drug-in-vesicle-in-gel system than in the insulin-loaded hydrogel without the use of vesicles [57].

Similar to chitosan and PMAA, polyacrylic acid (PAA) also contains a carboxyl group; this equips it with a pH-sensitive swelling property, thus making it a suitable polymer for incorporation into the hydrogel network [58]. PAA was grafted onto bacterial cellulose (BC) by means of electron beam radiation, to make use of the robustness of BC in supporting PAA-based hydrogels that are mechanically weak and biodegradable. As expected, the bacterial cellulose-*g*-poly(acrylic acid) [BC-g-P(AA)] hydrogel was pH-responsive, as the amount of insulin released in the first 2 h of incubation in the simulated gastric fluid was < 10%, but that in the simulated intestinal fluid was 77%–89% in 5–6 h, depending on the ratio of the hydrogel components. The BC-g-P(AA) hydrogel also reversibly reduced the transepithelial electrical resistance test values, with the effect being greater across the Caco-2 monolayer than across the Caco-2/HT29-MTX monolayer, possibly due to mucus. This was also reflected by the fact that the permeability of insulin increased by 3.5- to 5.9-fold, as compared to that of the control, which was an insulin solution. In an in vivo study in diabetic rats, a significant hypoglycemic effect was observed, with up to a 42%–49% reduction in blood glucose levels at 4–5 h for different compositions, as opposed to the negligible effect of oral insulin. The relative bioavailability ranged from 6.98% to 7.45%, in contrast to that of the oral insulin solution (0.64%). The viability of V-79, Caco-2, and HT29-MTX cells remained above 90% for all concentrations of the prepared hydrogels, although there was a downward trend as the concentration increased. Histological examination revealed no pathological changes in the intestinal or stomach tissues [58].

Carboxymethyl cellulose is another type of cellulose grafted with PAA [59]. Acrylate-grafted carboxymethyl cellulose (CMC-g-AA) also showed a pH-selective insulin release profile, with less than 10% released after 2 h at pH 1.2, and 80.8%–95.8% released after 6 h at pH 6.8. The percentage decreased with an increase in the percentage of CMC content and a decrease in the AA content, as it was speculated that a decrease in the percentage of AA could reduce the swelling of the hydrogel, resulting in a lower diffusion rate. In vivo studies in diabetic rats showed a 27.6% decrease in fasting blood glucose levels at 6 h after intragastric administration of the hydrogel loaded with 60 IU/kg insulin, which remained significantly lower than that of the oral insulin solution at 8 h. The relative pharmacological availability was 6.35% compared to that of the oral insulin solution, which was < 0.5%. MTT assay performed on CMC-g-AA hydrogels with different concentrations up to 1000 mg/L demonstrated a cell viability of > 90% [59].

The preclinical studies related to hydrogel formulations are summarized in Table 4.

**Table 4:** Preclinical studies on hydrogel formulation of oral insulin

Hydrogel formulation	Effect on the blood glucose levels in animals	Relative bioavailability (%)	Safety profile	Reference
Non-thiolized PCP hydrogel	Reduction by 30% in 2 h	1.95	Non-cytotoxic below 10 mM	[50]
Cys-PCP hydrogel	Reduction by 40%, and sustained for 8 h	2.45	Not available	[51]
P(MAA-g-EG) hydrogel	Reduction by 40% for ≥ 8 h	2.44–7.4	Not available	[52, 53]
PAA/S-chitosan hydrogel	Reduction for ≥ 6 h	4.43	No hepatotoxicity and nephrotoxicity	[54]
CMCD-g-CMC hydrogel	Reduction for 12 h, with peak at the 6th hour	Not available	Cell viability > 96%	[55]
SDS/β-CD vesicles-chitosan hydrogel	Not available	Not available	Not available	[57]
BC-g-P(AA) hydrogel	Reduction by 42%–49% in 4–5 h	6.98–7.45	Cell viability > 90% and no histological changes	[58]
CMC-g-AA hydrogel	Reduction by 27.6%	6.35	Cell viability > 90%	[59]

### Solid oral dosage form

NN1952 and insulin 338 (I-338) are two oral insulin formulations that use different GI permeation enhancement technologies (GIPETs) developed by Merrion Pharmaceuticals. NN1952 consists of fast-acting insulin 106, which is modified from human insulin by substituting Tyr-α14 and Phe-β25 with glutamine and histidine, respectively, and omitting Thr-β30, to reduce the susceptibility of insulin to gastric acid denaturation and proteolytic enzyme action [60]. Insulin 106 is tableted into GIPET-I, an enteric-coated tablet of insulin and medium-chain fatty acids in specific ratios [61], to increase the oral bioavailability of the otherwise poorly permeable insulin. I-338 is a long-acting basal insulin analog with sodium caprate as the absorption enhancer that modulates the tight junctions between epithelial cells and fluidity of the cell membranes [60, 62]. Similar to N1952, I-338 has been tableted into GIPET-I, with the insulin in it acylated by linking it to an 18-carbon fatty acid Table 5.

**Table 5:** Clinical studies on oral insulin

Type of formulation	Oral insulin formulation	Clinical trial reg. no	Development phase	Efficacy endpoint	Result	Safety profile	Reference
Liposomal nanoparticles	oral HDV-I	NCT0052178	Phase II	AUC of glucose concentration–time graphs	Significant decrease for the AUC_0–810 min_ period compared to placebo	● No serious adverse events ● No hypoglycemic events ● 3 treatment-emergent adverse events (right and left forearm IV infiltrate, right forearm IV site tenderness)	[74]
				Incremental AUC for plasma glucose	● Significant reduction at both 2 and 4 h post-breakfast compared to placebo ● No dose response effect		
Solid oral dosage form	NN1952	NCT01028404	Phase I	Area under the serum insulin concentration–time curve after a single dose	Compared to SC insulin: ● Higher insulin exposure ● Similar T_max_ ● Bio-efficacy = 0.7%	● Hyperglycemic and hypoglycemic events ● Diarrhea and hyperhidrosis possibly and probably related to NN1952	[76]
				Area under the glucose infusion rate-time curve after a single dose	● Maximum GIR of highest dosage (14,400 nmol) is comparable to SC insulin ● No significant difference in GIR compared to placebo		
	I-338	NCT02470039	Phase II	Change in fasting plasma glucose (FPG)	No significant difference compared to insulin glargine (-2.4 mmol/L vs -2.6 mmol/L)	● Similar number of adverse events ● Low incidence of hypoglycemia	[62, 77]
				Change from baseline in 10-points plasma glucose profile	No significant difference compared to insulin glargine		
				Change in HbA1C	No significant difference compared to insulin glargine		
	ORMD-0801	NCT02954601	Phase IIa	Changes in glucose levels from baseline to the end of treatment	● Greatest mean change in TDS dosing, followed by OD dosing ● Mean change in placebo is greater than BD dosing	Dose-dependent hypoglycemic events	[78]
		NCT02496000	Phase IIb	Mean change from baseline in weighted mean night-time glucose levels	● Significant decrease in 16 mg group compared to placebo ● No dose-dependent response	● 3 hyperglycemic events in 16 mg group ● Similar proportion of non-nocturnal hypoglycemic events and adverse events across all groups	[79]
		NCT03467932	Phase II	Change in HbA1c from baseline to Week 12	Significant reduction in 32 mg OD and BD dosing but not TDS	● Dose-dependent hypoglycemic events ● All hypoglycemic events occurred in patients on sulfonylureas ● Similar number of adverse events across all groups	[63, 81]
				Mean HbA1c change from baseline over time	8 mg OD or BD dosing demonstrated greatest mean change (at 0.95%)		
		NCT01889667	Phase II	Mean night-time glucose levels	Lowest in 8 mg BD, followed by 8 mg + 16 mg and placebo	No serious adverse events	[82]
				Mean daytime glucose levels	Similar trend as night-time levels		
	Eligen® insulin	NCT00982254	Phase I	Plasma insulin level	Significantly higher C_max_ and shorter T_max_ than SC regular insulin	No adverse events	[67]
				GIR	● Max relative BA: 26% ● Max biopotency: 55%		
	Capsulin	-	Phase II	GIR	● Actrapid showed significantly greater C_max_ and AUC_0–6 h_ ● Increase in GIR sustained for 6 h	No hypoglycemic or hyperglycemic events	[70]
				Plasma insulin level	Slight increase in both 150 U and 300 U Capsulin that is significantly lower than Actrapid		
Modified insulin	IN-105	CTRI/2009/091/000479	Phase II	Plasma glucose concentration under fed conditions	● Significant decrease across all doses (10, 15, 20, and 30 mg) compared to placebo ● No significant differences between 15, 20, and 30 mg dose	● 6 hypoglycemic cases and 1 case of relative hypoglycemia ● Most common adverse events: Increased serum triglyceride level ● No serious adverse events	[71]
				Plasma insulin level	Significantly proportional to dose		
	HIM-2	-	Phase II	Postprandial plasma glucose concentrations	Significant difference in glucodynamic parameters in 0.5 and 1.0 mg/kg dosing groups compared to placebo	● No serious adverse events ● No hypoglycemic or hyperglycemic event	[73]

*AUC* Area under curve, *BA* Bioavailability, *C*_*max*_ Maximum plasma concentration, *GIR* Glucose infusion rate, *T*_*max*_ Time to reach maximum plasma concentration

Enteric-coated oral insulin capsules (ORMD-0801) have been used to treat both type-1 and type-2 diabetes mellitus. The capsule also contains a soybean trypsin inhibitor, disodium ethylene-diamine tetraacetic acid, which improves absorption across the intestinal epithelium; Aerosil 200 as a stabilizer; and Tween 80 as a surfactant [63]. Preclinical studies on ORMD-0801 have been conducted in pigs and dogs. Pig models with intestinal access that bypasses gastric digestion were used, and ORMD-0801 lowered the AUC of blood glucose levels by 7.0%–7.5% in them [64]. ORMD-0801 has also been used with and without glucagon-like peptide 1 to control postprandial glucose. Upon administering enteric-coated capsules directly into the duodenum of the pigs, ORMD-0801 suppressed blood glucose levels for a portion of the monitoring period, in contrast to the increase observed in the group that received the placebo [65].

The pharmacokinetic and pharmacodynamic (PK/PD) profiles upon oral administration of ORMD-0801 were investigated in non-diabetic, healthy beagle canines and compared to those obtained upon with duodenal administration and treatment with insulin through the SC route. The maximum exogenous insulin concentration was highest for oral ORMD-0801. The T_max_ for oral ORMD-0801 was 0.75 h, while those for duodenally administered ORMD-0801 and SC insulin were 0.5 and 0.38 h, respectively. The mean AUC of insulin upon oral administration was similar to that obtained for duodenal administration of ORMD-0801, which was greater than that of SC insulin. The mean relative bioavailability was 5.41%, and the onset of action was 15 min after administration [66].

To overcome one of the greatest barriers to oral insulin absorption, that is, GI permeability, Eligen^®^ technology has been employed, where insulin is non-covalently complexed with a permeation enhancer such as monosodium N-(4-chlorosalicyloyl)-4-aminobutyrate (4-CNAB), which allows this macromolecule to be transported across the epithelial cells without causing histological damages or changes such as the opening of tight junctions [67–69]. Capsulin is another formulation that can improve the permeation of insulin through enteric coating and incorporation of insulin, aromatic alcohols, and a dissolution aid [70]. This formulation allows rapid dissolution upon contact with the intestinal epithelial lining.

### Modified insulin

Two distinct products have been created by altering the unbound amino acid on the Lys-β29 residue of synthetic human insulin. The addition of a small hydrophilic oligomer to the structure of insulin enhances its solubility and stability. This is due to the hydrophilic nature of the additional oligomer and the steric hindrance it creates, which prevents the enzymes in the GI tract from binding to insulin.

The oral tablet form of insulin analog IN-105 was chemically modified by attaching a methoxy triethylene glycol propionyl unit to the Lys-β29 residue of recombinant human insulin using a non-hydrolyzable amide bond [71, 72]. The solubility of the formulation was enhanced by adding PEG, which readily dissolves in water. Alternatively, hexyl insulin monoconjugate 2 (HIM-2), which involves the formation of a covalent connection between the Lys-29 of insulin and an amphiphilic oligomer, was collaboratively developed by Nobex Corporation and Biocon [73]. This solid oral dose, enclosed in a hard gelatin capsule, can inhibit the breakdown of proteins and improve their absorption in the intestines.

## Clinical studies

### Liposomal nanoparticles

#### Oral HDV-1

A single-blind, placebo-controlled clinical study sponsored by Diasome Pharmaceuticals was performed on six patients with T2DM using stable oral antidiabetic medicines. This study examined the relationship between the amount of oral HDV-I administered as a single dose before breakfast, lunch, and dinner and the resulting postprandial plasma glucose levels. The main objective of this study was to assess postprandial glycemic control, by analyzing the AUC of glucose concentration–time graphs. The secondary outcomes were the incremental AUC for plasma glucose and safety profile of the treatment. The most significant increase in blood glucose levels occurred after breakfast, followed by that after dinner, with the smallest increase observed after lunch. In summary, the findings indicated that the addition of oral HDV-I therapy at the specific doses examined (0.05, 0.1, 0.2, and 0.4 U/kg) led to a substantial decrease in the average postprandial plasma glucose AUC values for the AUC_0–810 min_ period, as compared to that observed in the placebo. The administration of all four doses of HDV-I resulted in a significant reduction in the average incremental AUC of plasma glucose at both 2 and 4 h after breakfast, as compared to that observed in the placebo. Nevertheless, administering all four doses of oral HDV-I did not result in a noteworthy decrease in the incremental plasma glucose AUC values, when compared to that observed in the placebo at either 2 or 4 h after lunch and dinner. The underlying cause for these effects was, however, not examined. Upon comparing the mean incremental glucose AUC values for the doses of oral HDV-I, a statistically significant difference was seen only between the 0.1 and 0.2 U/kg doses. Thus, it may be inferred that there is no established linear relationship between the dosage of a glucose-lowering medication and its effectiveness within the range of doses examined. Additionally, the lowest effective dose within this range was 0.05 U/kg. The safety profile of oral HDV-I was thoroughly examined, and no unforeseen or severe adverse events or hypoglycemic episodes were documented throughout the research. Nevertheless, four unfavorable incidents were observed in three individuals during placebo medication administration. These included two instances of headache, one case of itching in the left ear, and one case of hyperglycemia. One of the participants encountered three negative occurrences after HDV-I 0.4 U/kg treatment, namely, infiltration of the intravenous in both the right and left forearms and soreness at the intravenous injection site in the right forearm [74].

Another randomized, double-blind Phase II and III clinical trial sponsored by Diasome Pharmaceuticals compared the reduction in mean glycated hemoglobin levels between two doses of oral HDV-I and placebo in patients with T2DM on background metformin therapy for 18 weeks post-treatment, along with several other parameters, such as fasting plasma glucose, insulin, and frequency of hypoglycemic events [75]. The results of this study are yet to be published.

### Solid oral dosage form

#### NN1952

A safe dose of NN1952 with adequate pharmacodynamic response was identified in healthy subjects, and subsequently studied in patients with T2DM and compared to oral placebo and insulin aspart in a randomized, double-blind Phase I clinical study. In Part 1 of the study, six ascending doses (300, 900, 1800, 3600, 7200, and 14400 nmol) were administered to six different subjects, an oral placebo was administered to two subjects, and 162 nmol of insulin aspart was administered to the remaining two subjects. In Part 2, the patients with T2DM received a sequence of four treatments on separate days: NN1952 at the safe and appropriate dose identified in Part 1, with a 12-h euglycemic glucose clamp; insulin aspart with glucose clamp; NN1952 15 min before meals; and oral placebo 15 min before meals; with washout periods between each visit day, to minimize carry-over effects. Glucose clamps were used under fasting conditions to study their relative bio-efficacy and bio-availability, as compared to those of insulin, whereas prandial treatments were used to study postprandial glucose control under natural conditions. In the first part of the study, the highest dosage, which is 14400 nmol of NN1952, was chosen as the AUC_0–12 h_ for the glucose infusion rate (GIR), and the maximum GIR at this dosage was comparable to that of insulin, while being safe and well tolerated. Under fasting conditions, 14400 nmol of NN1952 induced a higher exposure of insulin, as compared to SC insulin, as reflected in the AUC_0–12 h_, and achieved its C_max_ at a similar time, which is at 1.5 h, as compared to that of 1.63 h observed in case of SC insulin, thereby showing rapid absorption. With respect to GIR, there was no significant difference between the AUC_0–12 h_ of both the treatments in the subjects with T2DM. Based on this AUC, the bio-efficacy was determined to be 0.7%. However, as compared to those of the placebo group, the prandial treatment groups showed no significant difference in the postprandial blood glucose levels, as estimated from the AUC of the blood glucose levels. The meal test also revealed large variability in the pharmacokinetic profile, which resulted in hyperglycemic and hypoglycemic events. Although there were no serious adverse events or deaths, two adverse events, namely diarrhea and hyperhidrosis, were recorded as possible and probably related to NN1952 [76].

#### I-338

One completed Phase I and Phase II clinical trial each have been conducted for I-338. A randomized, double-blind Phase I clinical trial sponsored by Novo Nordisk A/S determined the number of treatment-emergent adverse events recorded from the day of administration to the 12th day of the dosing visit [60]. In addition, an experiment was performed to determine the area under the serum insulin concentration–time curve on the 10th day. However, the results of this trial have not been published.

The effect of an 8-week treatment with a once daily dose of I-338 on fasting plasma glucose concentration, as compared to that of SC insulin glargine, was studied in a randomized, double-blind Phase II clinical study with the same sponsor. The trial also studied other parameters as secondary endpoints, such as 10-point plasma glucose concentration profiles and HbA1c levels. Fifty participants were randomized into the I-338 and insulin glargine groups. All other oral antidiabetic drugs, except metformin and DPP-4 inhibitors, were discontinued during a 2-week run-in period before the study was initiated. Upon comparing the I-338 and insulin glargine groups, there was no significant difference in the estimated mean change in fasting plasma glucose concentrations between the start of treatment and the end, with the levels dropping by 2.4 and 2.6 mmol/L, respectively. This was applicable to the mean plasma glucose concentration and HbA1c, which did not show any significant differences between the two groups, although a significant reduction in HbA1c could have been observed in the insulin glargine group with a larger sample size. This study demonstrated that I-338 is a promising candidate for oral basal insulin, comparable to the established insulin glargine. With respect to the safety profile, the number of adverse events was similar in both groups, with a low number of hypoglycemic events [62, 77].

#### ORMD-0801

Four Phase II clinical trials on ORMD-0801 are discussed below.

A single-center Phase IIa trial randomized 31 patients with diabetes into a treatment sequence of three treatment periods. For the three treatment periods, the participants received a placebo and one of the three active doses of ORMD-0801 once daily, twice daily, or thrice daily, for 5 d. Continuous glucose monitoring, which tracks changes in glucose levels from baseline to the end of treatment, served as the primary endpoint. ORMD-0801 thrice daily was associated with the greatest mean change in glucose levels, by –11.42 mg/dL, followed by once daily dosing, by –10.00 mg/dL. However, patients taking the fish oil placebo displayed a greater mean change in glucose levels than patients in the ORMD-0801 twice daily dosing group (–4.94 *vs.* –1.21 mg/dL, respectively). Although the specific doses used were not stated, investigation of the safety profile and tolerability of ORMD-0801 in patients with diabetes revealed that thrice daily doses of ORMD-0801 resulted in the highest incidence of hypoglycemic events, with five events across 21 participants; followed by twice daily dosing, with four events across 21 participants; and once daily dosing, with two events across 20 participants. In comparison, the placebo group recorded three hypoglycemic events across the 31 participants. Therefore, the incidence of hypoglycemic events was deduced to be dose-dependent [78].

Eldor, et al. [79] investigated the effect of ORMD-0801 on patients with T2DM who were treated with metformin for at least 2 weeks, where antidiabetic drugs other than immediate-release metformin were washed out for at least 14 d [80]. This was followed by a 2-week single-blind placebo run-in period. Subsequently, the patients were randomized in equal ratios to receive a placebo, ORMD-0801 16 mg insulin, or ORMD-0801 24 mg insulin at bedtime, at least 2 h after the evening meal, for 28 consecutive days. After 4 weeks, the mean nighttime glucose increased by 5.1% and 8.5% in the ORMD-0801 24 mg and placebo groups, respectively. This change was measured using continuous glucose monitoring carried out 6 h after treatment for two nights and compared with the baseline. However, the ORMD-0801 16 mg group showed a significant decrease (1.3%). In addition, the mean increase from baseline HbA1c levels was significantly lower in the ORMD-0801 16 mg group than in the placebo group. In the ORMD-0801 24 mg group, HbA1c levels decreased by 0.04. As no obvious dose–response was observed with ORMD-0801 (16 mg compared to 24 mg), it can be theorized that a 16 mg bedtime dose is the minimally effective dose in patients on metformin. Four treatment-emergent hyperglycemia events were reported in four individual patients, three of whom were administered ORMD-0801 16 mg, while one was administered a placebo. In each group, the patients experienced a non-nocturnal hypoglycemic event. Overall, a similar proportion of patients experienced one or more adverse events across all treatment groups [79].

Several clinical trials have been conducted to determine the optimal dosing regimen for ORMD-0801. In a study sponsored by Oramed Pharmaceuticals, diabetic patients on a stable dose of up to two oral antidiabetic medications for at least 3 months were recruited separately into two cohorts. In Cohort A, the participants were randomized to receive the following: placebo, ORMD-0801 32 mg once daily (at bedtime), twice daily (at bedtime and 30–45 min before breakfast), or thrice daily (at bedtime and 30–45 min before breakfast and lunch). There were 2 weeks of dose escalation periods for this cohort, where dosages were increased from 16 to 24 to 32 mg at their respective frequencies, followed by 10 weeks of stable doses. In Cohort B, the effects of smaller dosages were investigated. The participants received a randomized regimen of ORMD-0801 8 or 16 mg or a matched placebo, administered once or twice daily (at bedtime and 30–45 min before breakfast). In all treatment groups, there was a reduction from baseline HbA1c levels at 12 weeks, where the groups receiving ORMD-0801 32 mg once daily and twice daily had the highest reduction from baseline *versus* placebo (approximately –0.63 and –0.64, respectively, *vs.* –0.10; *p* = 0.04). However, the group administered a dosing regimen of 32 mg thrice daily experienced a smaller reduction in the least-squares mean of the change from baseline HbA1c levels (–0.55; *p* = 0.09). Hence, it can be concluded that, with respect to the reduction in HbA1c levels at 12 weeks, there is no significant advantage to dosing more than once daily in this group of patients. Overall, the least-squares mean change in HbA1c (%) from baseline to week 12 was greatest (at –0.95%) in Cohort B participants, who received ORMD-0801 8 mg once and twice daily. Cohort A participants, who underwent a dosing regimen of ORMD-0801 32 mg once and twice daily, followed this, with changes of –0.60% and –0.59%, respectively. Cohort B participants on a stable dose of ORMD-0801 (16 mg once daily) demonstrated a positive increase in the least-squares mean change in HbA1c (at –0.12%), as compared to the placebo (at –0.13%). Therefore, it can be concluded that a fixed, stable dosing regimen of ORMD-0801 8 mg once or twice daily is more beneficial in reducing 12-week HbA1c levels than a dose escalation regimen of ORMD-0801 or placebo [63, 81].

Dose-dependent hypoglycemic events were observed in this study. Thirty-two events of mild hypoglycemia events occurred in six patients who were administered ORMD-0801 (32 mg thrice daily), 23 of which occurred in a single patient. In comparison, 15 mild hypoglycemic events and one moderate hypoglycemic event occurred in six patients who were administered ORMD-0801 once daily. However, it should be noted that all events of hypoglycemia occurred in patients who were concurrently taking sulfonylureas. As for other adverse events, the occurrences were similar in the treatment and placebo arms, and most were mild or moderate. The most common adverse events were infections, and the majority were nasopharyngitis and GI disorders such as diarrhea and abdominal pain [63, 81].

A smaller study in 30 patients with T2DM examined the safety and PK/PD profile of taking multiple oral doses of ORMD-0801 before bed. Eligible participants included adults with poor glycemic control despite being treated with diet and exercise or diet, exercise, and metformin. The participants were randomized into three arms: ORMD-0801 8 mg + 8 mg, ORMD-0801 8 mg + 16 mg, and placebo oil capsules at bedtime. Studied as one of the secondary endpoints, the mean night-time glucose levels were lowest in those receiving ORMD-0801 8 mg + 8 mg (at 139.73 mg/dL), followed by those receiving ORMD-0801 8 mg + 16 mg (at 149.38 mg/dL), as compared to those receiving placebo oil capsules (at 165.85 mg/dL). A similar trend was observed for mean daytime glucose levels. Based on these early results, ORMD-0801 may lower HbA1c and mean glucose levels in people with T2DM who are not under control, even if they are taking metformin or other oral antidiabetic drugs [82]. Current data from published clinical trials suggest that ORMD-0801 is safe and well tolerated by most patients, including those who are antidiabetic naïve or those taking metformin and/or other oral antidiabetic agents. No mortality was reported in any of the aforementioned clinical studies.

However, despite positive results in the Phase II trials, two Phase III studies were terminated based on the primary results analyzed at the end of the treatment, and one Phase III study that was completed did not meet its primary efficacy endpoint, as per the press release from the sponsor company, although the study results were not published [83–86]. In this double-blind study, participants were randomly assigned to three groups, each receiving ORMD-0801 8 mg once daily, twice daily, or placebo for 26 weeks. The participants were expected to undergo another 26-week treatment extension period, but the study was terminated based on the primary results by week 26. The primary efficacy endpoint was the mean change in A1C from baseline at week 26, while the secondary endpoint was the mean change in fasting glucose levels [84].

#### Eligen^®^ insulin

One clinical study investigated oral insulin formulated using 4-CNAB. Its PK/PD properties were investigated using a euglycemic glucose clamp procedure for 6 h, in terms of plasma insulin levels and GIR. Ten male patients with T2DM were enrolled in this clinical trial and received either of the treatment arms: 300 U oral insulin combined with 400 mg 4-CNAB or 15 U regular SC insulin injection as a control on a separate dosing day. The C_max_ was significantly higher and T_max_ was significantly shorter with oral insulin administration than with SC regular insulin. In comparison to SC insulin, the maximum relative bioavailability of oral insulin was 26%, whereas the maximum biopotency was 55%; both were achieved at 0–1 h. No safety concerns were reported from this small study as no adverse events or clinically relevant changes in vital signs, electrocardiograms, or standard safety laboratory parameters were observed [67].

#### Capsulin

Similarly, the PK/PD profile of Capsulin has been investigated using an isoglycemic glucose clamp technique. The participants were randomized into two groups: Group 1 received 150 U Capsulin on Day 1, followed by 12 U SC Actrapid for the next 11 d, while Group 2 received Actrapid at the same dose on Day 1 and 300 U Capsulin for the next 11 d. During this treatment period, all oral hypoglycemic agents except metformin were stopped and replaced by 150 U Capsulin twice daily, administered 60 min before breakfast and the evening meal. All oral hypoglycemic agents were discontinued 12 h before the study day. On the study day, the GIR of dextrose was adjusted to maintain the basal plasma glucose concentration and used as a parameter to study the pharmacodynamic properties of Capsulin. In this study, Actrapid showed a significantly greater C_max_ and AUC_0–6 h_ for GIRs in both the groups, indicating greater glucose usage over 6 h. However, the GIR measured after Capsulin dosing in both the groups increased and was sustained for 6 h. It should be noted that the increase in plasma insulin levels following both Capsulin doses was minimal and significantly lower than that after insulin administration, as measured in terms of the AUC_0–6 h_. Despite the small increase in plasma insulin levels, the increase in GIR can possibly be attributed to the action of Capsulin, primarily in the liver, similar to that of natural insulin. Throughout the 10-day period between both the study days, when subjects were administered 150 U Capsulin twice daily, the blood glucose levels were controlled without any hypoglycemic or hyperglycemic events, despite the cessation of other oral hypoglycemic agents except metformin, thus indicating that Capsulin is safe and well tolerated [70].

### Modified insulin

#### IN-105

Twenty individuals with T2DM were included in a study funded by Biocon to compare IN-105 with a placebo. This study examined how each dose affected blood insulin, C-peptide, and glucose levels. The placebo was administered in the first period, followed by 10, 15, 20, and 30 mg IN-105 in each subsequent period, with a washout gap of at least 1 d to a maximum of 14 d between two subsequent periods. Overall, the plasma glucose levels decreased significantly across all doses of IN-105 administered, as compared to those observed with the placebo. After administration of 10, 15, 20, and 30 mg IN-105 tablets, the average maximum percent drop in glucose from baseline was 18.1%, 26.1%, 29.0%, and 30.8%, respectively. There were no significant differences between the effects seen with the 15, 20, and 30 mg doses. This linear dose–response relationship was also observed for the duration of glucose below baseline, average change in glucose 2 h postprandially, and AUC, in addition to the total plasma insulin level, where the C_max_ and AUC_0–t_ were significantly different across all doses and placebo groups. As the dose increased, six cases of hypoglycemia occurred in five participants, between 30 and 60 min after IN-105 was administered. One case of relative hypoglycemia after administration of 30 mg IN-105 required rescue with oral glucose, even though the blood glucose level was above 150 mg/dL, possibly because the blood glucose levels dropped quickly. The most common adverse event was increased serum triglyceride levels and no serious adverse events were reported [71].

Phase I studies have examined how IN-105 affects the oral absorption of metformin and its excipient, sodium caprate, which makes metformin more bioavailable. These studies also examined the best time to administer IN-105 in relation to meals [72, 87]. IN-105 can be administered in combination with metformin. Providing IN-105 10 or 20 min before meals can maintain high plasma insulin levels after a meal while preventing glucose levels from rising too quickly, resulting in a better PK/PD profile. This suggests that the co-administration of IN-105 with metformin and timed administration before meals can effectively manage postprandial glucose levels.

#### HIM-2

A randomized, single-blind, placebo-controlled, three-way crossover dose-escalation study sponsored by Nobex Corporation studied the efficacy of HIM-2 on postprandial glucose levels in patients with T2DM, in addition to its safety profile. The subjects were enrolled in one of the three dosing groups: 0.375, 0.5, and 1.0 mg/kg. In each group, oral HIM-2, insulin, and placebo were administered for three separate days. Higher doses of HIM-2 were allowed only when a lower dose was proven to be safe. Overall, the groups treated with 0.375 mg/kg HIM-2 and placebo showed no significant differences in all the parameters measured, including 2 h of postprandial glucose level and excursion, maximum plasma glucose concentration, and AUC_0–240 min_ of plasma glucose level. In contrast, HIM-2 was more effective than the placebo at controlling plasma glucose concentrations, at dosages of 0.5 and 1.0 mg/kg. No serious adverse events or incidences of hypoglycemia or hyperglycemia were reported, with the most reported adverse events being headache and anemia [73].

## Conclusion

The current discussion in T2DM therapeutics revolves around the difficulties in creating convenient oral insulin with minimal risk of hypoglycemia. Several approaches have been explored to address these challenges, such as nanoformulations, microemulsions, hydrogels, tableting, encapsulation, and modification of insulin through enteric coating, absorption enhancers, and enzyme inhibitors. Most of these formulations have exhibited effectiveness in the in vivo phase of preclinical investigations, as compared with the negative (placebo) and positive (SC insulin) controls. Additionally, these formulations have been proven to be safe in the cytotoxicity tests. The formulations that advanced to clinical trials yielded varied outcomes, with the bulk of the documented trials concluding in Phase II. To thoroughly assess the effectiveness and safety of these formulations in patients with T2DM, more Phase III trials must be conducted to establish their suitability in a wider population.

## Data Availability

No datasets were generated or analysed during the current study.
